# Computational fluid dynamics modelling of hemodynamics in aortic aneurysm and dissection: a review

**DOI:** 10.3389/fbioe.2025.1556091

**Published:** 2025-03-21

**Authors:** Mengqiang Hu, Bing Chen, Yuanming Luo

**Affiliations:** ^1^ State Key Laboratory of Transvascular Implantation Devices, Hangzhou, China; ^2^ Department of Technology, Boea Wisdom (Hangzhou) Network Technology Co., Ltd., Hangzhou, China; ^3^ The Second Affiliated Hospital of Zhejiang University, Hangzhou, China; ^4^ Department of Mechanical Engineering, The University of Iowa, Iowa City, IA, United States

**Keywords:** hemodynamics, CFD, aortic aneurysm, aortic dissection, wall shear stress

## Abstract

Hemodynamic analysis based on computational fluid dynamics (CFD) modelling is expected to improve risk stratification for patients with aortic aneurysms and dissections. However, the parameter settings in CFD simulations involve considerable variability and uncertainty. Additionally, the exact relationship between hemodynamic features and disease progression remains unclear. These challenges limit the clinical application of aortic hemodynamic models. This review presents a detailed overview of the workflow for CFD-based aortic hemodynamic analysis, with a focus on recent advancements in the field. We also conducted a systematic review of 27 studies with large sample sizes (n > 5) that examine the hemodynamic characteristics of aortic aneurysms and dissections. Some studies identified consistent relationships between hemodynamic features and disease progression, reinforcing the potential for clinical application of aortic hemodynamic models. However, limitations such as small sample sizes and oversimplified patient-specific models remain. These findings emphasize the need for larger, more detailed studies to refine CFD modelling strategies, strengthen the connection between hemodynamics and diseases, and ultimately facilitate the clinical use of aortic hemodynamic models in disease management.

## 1 Introduction

Aortic diseases, particularly aneurysms and dissections, remain significant global health threats. Between 1990 and 2019, fatalities from aortic aneurysms increased by 82.1% (Wang Z. et al., 2022). The true incidence of aortic dissection may be underestimated, as many patients die before receiving a diagnosis. Evidence also suggests that the number of aortic dissection cases has risen over time (LeMaire and Russell, 2011).

Currently, imaging is the primary method for diagnosing aortic aneurysms and dissections, providing anatomical parameters that guide therapeutic protocols. However, relying solely on anatomical risk assessments may overlook high-risk patients. For instance, while abdominal aortic aneurysms with a diameter over 5–5.5 cm or a growth rate above 1 cm per year typically prompt surgical intervention, some smaller aneurysms can also be prone to rupture (Spanos et al., 2020; Abdelkhalek et al., 2022; Ashkezari et al., 2022). Similarly, certain type B aortic dissections (TBAD) that appear clinically stable may in fact be unstable, contributing to a persistent in-hospital mortality rate in recent years (Evangelista et al., 2018).

Recent studies increasingly suggest that hemodynamic factors could improve risk stratification for patients with aortic aneurysms or dissections (Zilber et al., 2021; Ramaekers et al., 2023). Aortic hemodynamics can be assessed through clinical measurements or *in silico* analysis based on computational fluid dynamics (CFD). Compared to clinical measurements, CFD modelling offers more precise spatiotemporal predictions at a lower cost. However, many clinicians face challenges in implementing CFD modelling and interpreting its results due to insufficient training in this area.

This review aims to present the recent advancements in CFD modelling for predicting aortic hemodynamics, providing guidance on conducting CFD simulations and analyzing hemodynamic indicators. We also reviewed several large sample studies to summarize findings on the relationships between hemodynamic metrics and aortic diseases, while highlighting limitations that currently hinder the clinical application of aortic hemodynamic models. Finally, we offer recommendations for future research on CFD-based hemodynamic analysis in aortic aneurysms and dissections.

## 2 Features of aortic hemodynamics

The aorta, the largest blood vessel in the body, serves as the primary conduit for delivering oxygenated blood throughout the body (Hall and Guyton, 2011). Hemodynamics within the aorta is highly complex, largely due to its unique anatomy. The aorta’s centerline is non-planar and curved in three dimensions (Chandran, 1993). As blood is ejected from the left ventricle, it undergoes impingement, swirling, separation, and reattachment as it navigates the tortuous path of the aorta. Additionally, the large size of the aorta leads to the development of secondary flows within the lumen cross-section (Tse et al., 2013).

The numerous branches of the aorta contribute to the redistribution of blood flow, adding to the complexity and uncertainty of the aortic hemodynamics. The aorta extends from the left ventricle to the lower abdomen, tapering in lumen size and branching multiple times along the way. It typically has three main branches in the aortic arch that supply blood to the upper body, as well as several branches in the descending aorta that deliver blood to internal organs. However, the branching patterns of the aorta exhibit considerable variability among individuals (Mustafa et al., 2017). Blood flow is redistributed at these bifurcations, and the presence of lesions, such as stenosis, further complicates the flow dynamics (Dabagh et al., 2015).

Significant changes in the shape and spatial position of the aortic vessel wall further contribute to the complexity of intraluminal hemodynamics. The aorta’s compliance is high, accounting for more than half of the total compliance of the arterial system in healthy young adults (Pagoulatou et al., 2021). The aorta stretches to accommodate the large volume of blood ejected during systole and recoils to maintain downstream blood flow during diastole (Tanweer et al., 2014). Additionally, the aortic root experiences periodical towed and relaxed movements due to ventricular traction, which are transmitted to the ascending aorta (Wei et al., 2019). Given these factors, predicting aortic hemodynamics through CFD modelling presents significant challenges.

## 3 Workflow in CFD modelling for aortic hemodynamics

Partial differential equations are commonly used to describe fluid flow and related phenomena. However, in many cases, these equations cannot be analytically solved. CFD modelling addresses this challenge by using discretization methods to approximate partial differential equations as a system of algebraic equations (Feiger et al., 2020). These algebraic equations are then solved through computational techniques. The obtained numerical solution provides results at discrete locations in both space and time.

Despite its potential, CFD modeling for aortic hemodynamics faces several challenges, including high computational demands, long processing times, and the complexity of the process, which requires multidisciplinary collaboration (Cebral and Meng, 2012; Feiger et al., 2020; Kamada et al., 2022). Additionally, the highly patient-specific nature of these simulations adds further complexity. However, the successful use of CFD modelling in cardiovascular diseases, such as noninvasive fractional flow reserve, has strengthened confidence in its broader clinical potential (Hu et al., 2024). Researchers remain committed to advancing CFD techniques for aortic diseases management, aiming to bridge the gap between research and clinical practice.

CFD modelling of hemodynamics involves seven stages: clinical imaging, geometry reconstruction, discretization, boundary condition specification, solution setup, post-processing, and validation (Morris et al., 2016).

### 3.1 Clinical imaging

Traditional clinical imaging methods for geometry reconstruction in hemodynamic studies include digital subtraction angiography (DSA), contrast-enhanced computed tomography (CECT), computed tomography angiography (CTA), magnetic resonance imaging (MRI), and magnetic resonance angiography (MRA) (Rayz and Cohen-Gadol, 2020). DSA is an invasive technique that provides two-dimensional projections of the vasculature. It can be expanded to three dimensions through 3D rotational angiography (3DRA), which acquires and combines multiple projections by rotating the C-arm. 3DRA is considered the gold standard for generating intracranial aneurysm (IA) models (Steinman and Pereira, 2019). CECT and CTA are the most commonly used imaging method, offering high spatial resolution and rapid acquisition times (Ren et al., 2016). However, all of these methods, including DSA, 3DRA, CECT, and CTA, involve ionizing radiation and nephrotoxic contrast agents. MRI is a technique free from ionizing radiation but requires longer acquisition times and offers lower spatial resolution (Wan Ab Naim et al., 2014). Unenhanced MRA techniques are noninvasive. Time-of-flight MRA is a representative unenhanced technique that uses repeated pulses to differentiate between stationary tissues and flowing blood. However, this technology performs poorly in regions with slow flow (François et al., 2008). This limitation can be addressed with contrast-enhanced MRA. In addition to lumen modelling, MRI can also provide valuable data on wall content through black blood MRI scans (Arzani et al., 2014). Wang et al. (2022) systematically reviewed the applications of MRI in the hemodynamic study of TBAD.

Moreover, ECG-gated imaging techniques have been employed to capture dynamic variations in the aortic wall position throughout the cardiac cycle. Capellini et al. (2021) reconstructed ascending aortic geometries from ECG-gated CTA data and examined how geometric variations influence aortic hemodynamics. Similarly, Midulla et al. (2012) used ECG-gated cine MRA to capture aortic wall movements and performed dynamic mesh simulations to predict hemodynamics in patients treated by thoracic endovascular aortic repair.

### 3.2 Geometry reconstruction

Based on the acquired image dataset, geometry reconstruction can proceed, involving several steps such as image segmentation, smoothing, simplification, and treatment of the inlet and outlet.

Segmentation methods have evolved from 2D plane segmentation and lofting to 3D volumetric segmentation based on intensity thresholding, and now to fully automated segmentation powered by deep learning (Steinman, 2002; Antiga et al., 2008; Cao et al., 2019; Chen et al., 2021). A state-of-the-art deep learning-based framework is able to identify the true and false lumen, as well as local characteristics of the primary tear, in patients with TBAD (Chen et al., 2021).

Typically, some degree of smoothing is applied to the segmented model to facilitate successful spatial discretization. However, this smoothing must be performed carefully to avoid altering the geometry and volume size. Paritala et al. (2023) investigated intra-team variability in hemodynamic predictions for IAs and found that smoothing contributed the most to variations in wall parameters. Nonetheless, its impact on aortic hemodynamics remains underexplored.

Early hemodynamic studies often employed some simplifications, such as neglecting variations in vessel diameter, using circular cross-sections, and omitting downstream branches (Caballero and Laín, 2013). These simplifications can significantly affect the accuracy of the hemodynamic predictions. As computational power improves, the use of complete, patient-specific aortic models is increasingly recommended (Wan Ab Naim et al., 2014). Stokes et al. (2023b) investigated the impact of neglecting minor aortic branches on TBAD hemodynamics and found that this simplification could lead to differences of 60%–75% in clinically relevant metrics.

Truncating and extending the inlet and outlet appropriately ensures that the blood flow in the region of interest is not affected by boundaries. These strategies are typically determined based on the research needs. While some studies have quantitatively assessed their impact on intracranial hemodynamics (Pereira et al., 2013; Hua et al., 2015), similar analyses on the aorta are relatively rare. Chi et al. (2022) highlighted the importance of appropriate inlet truncation by comparing the effects of truncating at the middle of the ascending aorta versus at the aortic valve.

Geometry reconstruction can be carried out using various software tools, including Mimics (Geiger et al., 2016), SimVascular (Updegrove et al., 2017), CRIMSON (Arthurs et al., 2021), VMTK (Williams et al., 2022), Simpleware ScanIP (Stokes et al., 2023a), 3D Slicer (Pieper et al., 2004), ITK-SNAP (Yushkevich et al., 2016), OsiriX (Rosset et al., 2004), GIMIAS (Larrabide et al., 2009), MITK (Wolf et al., 2004), DetecModeling (Ding et al., 2024). Some tools, such as SimVascular and DetecModeling, incorporate advanced machine learning algorithms, which streamline the reconstruction process and reduce the time and effort required.

To ensure that the reconstructed geometric model is suitable for CFD simulation, several measures should be taken (Sveinsson Cepero and Shadden, 2025). First, the research team should consist of experienced biomedical engineers familiar with human anatomy. A comprehensive geometric reconstruction protocol should be developed, covering key aspects such as intensity thresholding, smoothing extent, and inlet and outlet truncation and extension. All operators must undergo consistent training, and each reconstruction task should be performed by at least two operators. Any disagreements between operators should be resolved by experienced team members (Paritala et al., 2023). From a technical standpoint, advanced automated algorithms should be prioritized to reduce operator-induced uncertainties (Maher et al., 2020). Additionally, sequentially combining advanced algorithms–such as imaging quality enhancement, centerline extraction, and segmentation–can further improve the accuracy of the reconstruction process (Lesage et al., 2009; Maher et al., 2021).

### 3.3 Spatial discretization

Once the geometric reconstruction based on clinical images is completed, the process can proceed similarly to CFD simulations in other fields, starting with spatial discretization, or grid generation. The computational domain is typically discretized into tetrahedral, hexahedral, or polyhedral meshes using advanced software or algorithm. A finer boundary layer mesh should be generated in regions adjacent to the arterial wall to accurate predict near-wall flow and wall parameters. Mesh resolution significantly impacts the accuracy of hemodynamic predictions (Aycan et al., 2023). Although this has primarily been demonstrated in studies of IAs and coronary arteries (Prakash and Ethier, 2001; Hodis et al., 2012), it can be logically extended to the aorta. Moreover, the mesh resolution required for mesh independence varies for different hemodynamic parameters, such as velocity and wall parameters (Prakash and Ethier, 2001; Valen-Sendstad and Steinman, 2014; Evju et al., 2017), and should be carefully determined.

### 3.4 Boundary conditions

To obtain accurate hemodynamic predictions, it is essential to establish appropriate boundary conditions for the computational domain. Clinically measured, patient-specific data, such as blood flow velocity, pressure, and arterial wall movements, are considered ideal for hemodynamic simulations (Rayz et al., 2008; Perera et al., 2020); however, these data are often unavailable in clinical practice.

For the inlet boundary condition, the empirical flow waveform from literature is typically used in aortic hemodynamic simulations. However, studies have shown that deviations in the inlet flow rate, flow waveform shape, and inlet velocity profile can hinder the accurate prediction of patient-specific hemodynamics (Morbiducci et al., 2013; Youssefi et al., 2018; Armour et al., 2021; Stokes et al., 2023a). Armour et al. (2021) found that a reduction in stroke volume resulted in a decrease in both velocity and wall shear stress, whereas the flow waveform shape significantly affected the predicted pressure. Furthermore, their results also revealed that three inlet velocity profiles–three-dimensional, through-plane, and flat profiles–yielded similar predictions for velocity and wall shear stress; however, the through-plane profile predicted a more accurate pressure distribution. Similarly, Stokes et al. (2023a) found that the choice of the inlet velocity profile significantly influenced oscillatory shear and helicity in the aorta. Although 4D-flow MRI can measure the temporal three-dimensional velocity profile, it has been shown to underestimate the peak flow rate and velocity (Cherry et al., 2022). Therefore, 2D-flow MRI, which acquires axial velocity data (i.e., through-plane profile), is recommended for measuring patient-specific inlet boundary conditions (Armour et al., 2021; Stokes et al., 2023a).

For the outlet boundary condition, obtaining clinically measured data is more challenging due to the presence of multiple outlets and the small size of the downstream branches. In aortic hemodynamic simulations, simplified outlet boundary conditions, such as constant or pulsatile pressure (O’Rourke et al., 2012; Filipovic et al., 2013; Zambrano et al., 2016), or specific flow division (Peng et al., 2019; Zhang et al., 2023), are commonly used. However, the neglect of the downstream arterial system in simulations using these simplified boundary conditions often results in non-physiological predictions, particularly regarding blood pressure. Furthermore, prescribing equal pressure for all outlets causes the downstream flow distribution to deviate from reality (Chnafa et al., 2018). Coupling low-dimensional models, such as the lumped parameter model (0D) or distributed parameter model (1D), at outlets to perform multi-scale simulations is an effective option for predicting hemodynamics closer to physiological conditions (Soudah et al., 2014; Stokes et al., 2021). A comprehensive introduction to these low-dimensional models for hemodynamic predictions can be found elsewhere (Shi et al., 2011; Zhou et al., 2019; Chi et al., 2022; Garber et al., 2022).

For the aortic vessel wall, variations in the shape and spatial position due to compliance and cardiac traction are often ignored in simulations, which typically assume that the wall is rigid with no-slip boundary conditions (Xu et al., 2021; Li et al., 2023; Mutlu et al., 2023). However, the assumption of a rigid wall has been shown to cause significant deviations in the blood flow velocity and wall parameters in hemodynamic predictions (Ene et al., 2014; Bonfanti et al., 2017; Qiao et al., 2019; Athani et al., 2022). Ene et al. (2014) compared fluid-structure interaction (FSI) simulation using a linear elastic material model with rigid wall CFD simulation in a realistic abdominal aortic aneurysm. They found that the rigid wall CFD simulation overestimated both the velocity magnitude and wall shear stress. Bonfanti et al. (2017) developed a patient-specific CFD multi-scale approach incorporating aortic wall compliance and applied it to study the hemodynamics of aortic dissection. The results indicated that the transmural pressure predicted by the compliant model was higher than that predicted by the rigid model. The systolic flow rate in the true lumen predicted by the compliant model was lower than that predicted by the rigid model, while the diastolic flow rate in the true lumen predicted by the compliant model was higher. Additionally, differences in the magnitude and oscillation of the wall shear stress were observed between compliant and rigid models (Qiao et al., 2019). Wall compliance in aortic hemodynamics can be addressed using several approaches. For instance, Pant et al. (2014) proposed a lumped parameter method that introduced a capacitor before the inlet to account for FSI effects due to aortic compliance. Some studies have modeled the aortic wall as either linear or nonlinear (Alimohammadi et al., 2015; Bonfanti et al., 2018; Qiao et al., 2019; Kim et al., 2023), and isotropic or anisotropic (Jayendiran et al., 2018; Vignali et al., 2021; Wang et al., 2023) materials to perform FSI simulations. Additionally, based on aortic wall movements observed during clinical imaging, some researchers have utilized dynamic mesh methods (Ramiro et al., 2006; Midulla et al., 2012) or radial basis functions mesh morphing technology (Capellini et al., 2018; 2021; Calò et al., 2023) to perform FSI simulations. The impact of arterial wall elasticity on hemodynamic predictions varies significantly across different disease scenarios (Morab et al., 2024). Therefore, the influence of FSI simulations on the accuracy of aortic hemodynamics predictions requires further exploration. A comprehensive introduction to these FSI simulation approaches for hemodynamic predictions can be found elsewhere (Humphrey and Holzapfel, 2012; Hirschhorn et al., 2020; Athani et al., 2024).

### 3.5 Solution setup

Before performing CFD simulations, it is crucial to select the appropriate viscosity and turbulence models, as well as determine the time dependence and convergence criteria for the simulation.

The viscosity model characterizes the blood rheology. As a non-Newtonian fluid, the viscosity of blood varies with the shear rate. However, the assumption of Newtonian rheology is commonly adopted in hemodynamic studies because shear-thinning behavior is not significant in large arteries (Mutlu et al., 2023) and non-Newtonian simulations are more time-consuming (Liu et al., 2023). Qiao et al. (2019) compared the performance of a two-phase non-Newtonian model with the Newtonian model in predicting aortic dissection hemodynamics. They found that the Newtonian model might underestimate the magnitude of wall shear stress. However, Qiu et al. (2018) and Marrero et al. (2014) found that hemodynamic differences in aortic aneurysms due to the viscosity model were negligible. A comprehensive review of viscosity models for hemodynamic prediction can be found elsewhere (Yilmaz, 2008; Kannojiya et al., 2021).

The turbulence model is used to predict flow instability, which can be categorized based on the Reynolds number (Re) into laminar, transitional, and turbulent flow states. In straight, uniform tubes with steady flow, turbulence typically occurs when the Reynolds number exceeds the critical threshold of 2200 (Westerhof et al., 2019). However, for blood flow in the aorta, the pulsatile nature of the flow alters this threshold. Specifically, the descending phase of the cardiac cycle has been shown to facilitate the transition to turbulence (Winter and Nerem, 1984), while flow acceleration tends to have the opposite effect. The Womersley number (α), which quantifies the relative importance of inertial versus viscous forces, provides valuable insight into these oscillatory characteristics (Womersley, 1957). Nerem et al. (1972) confirmed that the occurrence of turbulence is related to both the peak Reynolds number (Rê) and the Womersley number in an experimental study conducted in the canine ascending and descending aorta. As a result, hemodynamic studies typically assess the likelihood of turbulence based on the position of the inlet flow regime on the Rê–α diagram (Kousera et al., 2013). Additionally, some researchers suggest that the tortuous nature of arteries, along with sudden contractions or expansions at lesion sites, can also induce turbulence (Katz et al., 2023). Les et al. (2010) investigated aortic hemodynamics in eight patients with abdominal aortic aneurysms using direct numerical simulation (DNS). They observed that turbulence was primarily present distal to the aneurysm. DNS does not rely on assumptions about laminar or turbulent flow, making it particularly suitable for modelling transitional arterial flow. However, its high computational cost limits its widespread application in hemodynamic research. As alternatives, large eddy simulation (LES), eddy-viscosity models, and Reynolds-stress models are commonly employed for turbulence modelling in aortic hemodynamics, as summarized in Table 1. Among these models, the transition shear stress transport (SST) turbulence model is considered the most promising Reynolds-averaged Navier-Stokes model for predicting laminar-turbulent transition in large arteries (Tan et al., 2009; Kousera et al., 2013). Based on this turbulence model, Cheng et al. (2014) successfully predicted blood flow pattens that closely matched those measured using phase-contrast MRI in a patient with aortic dissection.

**TABLE 1:** Turbulence models used in aortic hemodynamic simulations.

Reference	Study object	Turbulence model
Xu et al. (2018)	Type-B aortic dissection	LES model
Martínez et al. (2023)	Ascending aorta	
Rezaeitaleshmahalleh et al. (2024)	Abdominal aortic aneurysm	
Black et al. (2023)	Type-B aortic dissection	Standard k-omega turbulence model
Algabri et al. (2019)	Abdominal aortic aneurysm	SST k-omega turbulence model
Stokes et al. (2023a), Stokes et al. (2023b)	Type-B aortic dissection	
Kousera et al. (2013)	Healthy aorta	Transition SST turbulence model
Chi et al. (2017)	Type-A aortic dissection	
Cheng et al. (2014)	Type-B aortic dissection	
Cheng et al. (2015) Wan Ab Naim et al. (2018) Dai et al. (2020)		
Etli et al. (2021)	Thoracic aortic aneurysm	k-epsilon turbulence model
Moretti et al. (2023)	Aortic dissection	
Hohri et al. (2021)	Type-A aortic dissection	
Long Ko et al. (2017)	Type-B aortic dissection	
Botar et al. (2017)	Abdominal aortic aneurysm	Reynolds-stress model

For time dependence, transient CFD simulations are commonly employed in hemodynamic studies to capture the pulsatile nature of blood flow accurately. However, these simulations can be time-intensive, which has prompted some researchers to explore alternative steady-state simulations. Qiu et al. (2018) found that in an abdominal aortic aneurysm model, the time-averaged wall shear stress distribution in transient simulations closely matched that of steady-state simulations. Similar findings were reported by Chi et al. (2022) and Caballero and Laín (2015) for the thoracic aorta. Nonetheless, with the increasing availability of computational resources and advancements in parallel processing methods (Mirzaee et al., 2017; Hu et al., 2023), transient simulations have become the primary method for hemodynamic studies. These simulations allow for the calculation of various hemodynamic indicators over time, providing more comprehensive insights into flow dynamics. For transient simulations, the time step size plays a crucial role in the accuracy of predictions, as demonstrated in studies of IAs (Valen-Sendstad et al., 2013; Valen-Sendstad and Steinman, 2014; Dennis et al., 2017). Although similar investigations on the aorta are less common, it is recommended to perform a time-independence check before conducting transient simulations to ensure the accuracy of results.

Convergence criteria are also vital for ensuring that CFD simulations reach a valid endpoint. Dennis et al. (2017) found that a residual error of 10^–5^ for mass and momentum equations was sufficient for convergence in their IA model; however, this threshold may vary for different geometries. Additionally, for transient simulations, it is equally important to verify the statistical convergence of temporal indicators by comparing these values across at least two cardiac cycles (Poelma et al., 2015).

Several popular open-source and commercial CFD solvers are available for hemodynamic simulations (Azizi, 2023), with general-purpose software such as Ansys Fluent, Ansys CFX, COMSOL, OpenFOAM, and STAR-CCM+ being widely used. However, these general tools often require additional user programming to include specific cardiovascular functions, such as the Windkessel outlet boundary. In contrast, cardiovascular-specific software, including SimVascular (Updegrove et al., 2017), CRIMSON (Arthurs et al., 2021), and DetecFluid (Liu et al., 2025), are more streamlined for hemodynamic applications as they already integrate these functions. Moreover, in-house developed codes are also used for specialized hemodynamic analyses, such as DNS for turbulence (Les et al., 2010).

### 3.6 Postprocessing and related metrics

Upon completing the numerical solutions, fundamental physical quantities like velocity and pressure are determined within the computational domain. From these quantities, various hemodynamic metrics are derived to analyze and characterize blood flow behavior. These metrics include flow pattern descriptors such as vorticity and helicity, energy-related parameters such as energy loss and pressure loss coefficient, and wall shear parameters like wall shear stress (WSS) and oscillatory shear index (OSI). Some of these metrics, initially proposed for arterial vessels other than the aorta, can be extended to other vascular regions due to shared underlying mechanisms. For instance, Shimogonya et al. (2009) introduced the gradient oscillatory number (GON) as a metric to assess the risk of IA initiation. GON can also be used to evaluate the risk of aortic aneurysms, as both the intracranial and aortic aneurysms shear similar pathophysiological factors, such as endothelial dysfunction, inflammation, and remodeling of the arterial wall induced by hemodynamic factors (Tanweer et al., 2014; Signorelli et al., 2015; 2018; Gao et al., 2023). As such, a comprehensive summary of hemodynamic metrics relevant to both aortic and non-aortic diseases is provided in the Supplementary Table S1 online.

Certain hemodynamic metrics exhibit strong correlations with one another. For example, Lee et al. (2009) found significant correlations between relative residence time (RRT) and time-averaged WSS (TAWSS) or OSI, suggesting that RRT might serve as a robust single metric for identifying low or oscillating shear regions. Additionally, metrics vary in their sensitivity to CFD settings. Lassila et al. (2020) demonstrated that although both OSI and transverse WSS (transWSS) can be used to assess the directional variation of the WSS vector, OSI is more sensitive to changes in blood flow dynamics in IAs. Consequently, transWSS performed better in assessing the risk of IA rupture. This highlights the importance of selecting appropriate metrics based on specific research goal and simulation settings.

In terms of data visualization during postprocessing, various graphic representations are commonly employed, including contour plots, vector plots, streamline plots, and isosurfaces of hemodynamic metrics. Postprocessing is often conducted at different levels of data granularity, with two main categories of focus. The first category is concerned with specific lesion sites, such as the rupture point of an aortic aneurysm (Teng et al., 2022) or the primary entry tear of an aortic dissection (Chi et al., 2017). The second category focuses on broader hemodynamic characteristics, such as the maximum, minimum, and average values of metrics within the aneurysm dome (Bappoo et al., 2021; Wen et al., 2023).

### 3.7 Validation

To ensure that CFD modelling accurately predicts the hemodynamic characteristics of the aorta, validation efforts are essential, though they can be complex and costly. CFD validation typically involves three approaches: *in vitro* validation using an idealized model (Mansouri et al., 2024), *in vitro* validation with a patient-specific model created through 3D printing (Ong et al., 2019; Bonfanti et al., 2020), and *in vivo* validation based on clinical measurements (Bonfanti et al., 2017; Bonfanti et al., 2019; Boccadifuoco et al., 2018; Stokes et al., 2021). Table 2 provided a summary of these validation studies related to aortic hemodynamics, highlighting CFD settings, validation methods, validated parameters, and key findings. Among these methods, *in vivo* validation against clinical data is considered the most reliable way to confirm CFD accuracy. Typically, clinical studies utilize invasive blood pressure measurements taken by arterial catheter (Bonfanti et al., 2019), blood flow rates obtained via phase-contrast MRI (Bonfanti et al., 2017), and cross-sectional velocity profiles captured by 4D-flow MRI (Stokes et al., 2021) as reference values. However, other hemodynamic metrics, such as WSS, are more difficult to validate due to limitations in the temporal and spatial resolution of current measurement techniques (Miyazaki et al., 2017).

**TABLE 2:** Summary of validation studies on aortic hemodynamics.

Reference	Study object and geometry model	CFD settings	Validation methods, validated parameters, and major findings
Mansouri et al. (2024)	Healthy thoracic aorta; idealized aorta with major anatomic features	Steady-state, k-omega turbulence model, Newtonian fluid, rigid wall, uniform bulk velocity at inlet, zero-pressure outlet	PIV experiments; velocity and flow patterns. The global flow patterns in the idealized model showed excellent qualitative and quantitative agreement between CFD simulations and PIV experiments.
Ong et al. (2019)	Thoracic aortic aneurysm; patient-specific model	Transient, laminar, Newtonian fluid, compliant wall with linear elastic material, time-dependent flow profile at inlet, pressure waveform at outlet	PIV experiments; flow at inlet, pressure at outlets, and flow pattern. Flow behaviors observed in FSI simulations closely matched those in experiments.
Bonfanti et al. (2020)	Type B aortic dissection; patient-specific model	Transient, SST k-omega turbulence model, Newtonian fluid, rigid wall, flow rate waveform at inlet, three-element Windkessel model at outlet	PIV experiments; flow rate and pressure at inlet and outlets, velocity, flow patterns. Numerical results on flow rate, pressure, and velocity fields closely matched experimental data.
Bonfanti et al. (2017)	Type B aortic dissection; patient-specific model	Transient, laminar, non-Newtonian fluid (Carreau-Yasuda model), compliant wall (moving boundary method), uniform velocity profile at inlet, three-element Windkessel model at outlet	2D phase-contrast MRI; flow rate at different locations along the aorta, displacement of the vessel wall. CFD simulations underestimated peak flow rates, but accurately predicted flow waveforms and vessel wall displacement.
Miyazaki et al. (2017)	Healthy aortic arch and double aortic arch; patient-specific model	Transient, laminar or LES or k-epsilon model, Newtonian fluid, rigid wall, flow rate waveform at inlet, pressure waveform at outlet	4D-flow MRI; velocity, flow patterns, WSS distribution, energy loss. CFD simulations accurately predicted flow patterns distal to the aortic arch but failed to capture helical flow in the ascending aorta. WSS distribution matched MRI results, though the average WSS was five times higher, likely due to MRI’s low spatial resolution.
Boccadifuoco et al. (2018)	Healthy thoracic aorta; patient-specific model	Transient, laminar, Newtonian fluid, rigid or compliant wall (linear elastic material), flow rate waveform at inlet, three-element Windkessel model at outlet	4D-flow MRI; flow rate waveform at outlets, vessel wall displacement, velocity fields. CFD simulations showed discrepancies with measured results, particularly in the aortic arch and descending aorta. Using a lower Young’s modulus improved accuracy but tended to significantly underestimate peak flow rate.
Bonfanti et al. (2019)	Type B aortic dissection; patient-specific model	Transient, laminar, non-Newtonian fluid (Carreau-Yasuda model), rigid wall with a capacitor before the inlet to account for FSI effects, flow rate waveform at inlet, three-element Windkessel model at outlet	Invasive blood pressure measurements by catheter; pressure in the true and false lumen along the aorta. CFD predictions closely matched invasive catheter measurements.
Stokes et al. (2021)	Healthy thoracic aorta; patient-specific model	Transient, SST k-omega turbulence model, rigid wall or compliant wall (moving boundary method), flow rate waveform at inlet, three-element Windkessel model at outlet	Cine-MRI and 4D-flow MRI; flow rate waveform, luminal cross-sectional area variation, velocity, flow patterns. Simulated luminal area and flow rate variations closely matched clinical measurements. Compliant and rigid simulations over- and under-predicted peak flow rates, while velocity profiles showed moderate consistency with 4D-flow MRI data.

Furthermore, some validation studies have investigated the impact of CFD modelling settings on prediction accuracy. Boccadifuoco et al. (2018) compared the performance of rigid and compliant vessel wall models in predicting the hemodynamic characteristics of the thoracic aorta. By comparing their results with *in vivo* phase-contrast MRI data, they found that FSI simulations with a compliant wall yielded more accurate flow velocity predictions. The role of FSI simulations on the accuracy of hemodynamic predictions across various disease states requires further exploration (Morab et al., 2024). Additionally, Black et al. (2023) studied how parameters in three-element Windkessel models at the outlets affect the predicted flow rate waveforms. Their findings showed that using parameters calibrated with flow waveforms from 4D-flow MRI data resulted in more accurate predictions.

## 4 Studies and limitations

The primary goal of hemodynamic prediction is to establish a link between hemodynamic metrics and aortic diseases and their related complications. This understanding can inform better clinical management strategies. We conducted a systematic search to investigate the application of hemodynamic prediction in aortic aneurysm and dissection. A full description of the search and screening strategy can be found in the Supplementary Material online. To ensure a reliable connection between hemodynamics and aortic diseases, we focused on studies with sample sizes greater than five, including 17 studies on aortic aneurysms and 9 studies on aortic dissections.

Accurate correlation between hemodynamic features and diseases requires careful study design. Common methods used in these studies include virtual repair, longitudinal analysis, and parallel control (Peiffer et al., 2013). Virtual repair is often to identify risk factors associated with the occurrence of aortic diseases. However, due to the complex shape changes resulting from aortic aneurysms or dissections, there is still a lack of efficient, automated technology for repairing and modelling a healthy aorta. Longitudinal analysis is frequently employed to link hemodynamic metrics with disease progression. However, this approach is limited by the potential radiation exposure associated from clinical imaging. Parallel control offers the greatest flexibility and is applicable to all stages of aortic diseases, but it typically requires a large sample size to minimize the impact of intra-group differences.

Despite the increasing body of research on aortic hemodynamics, these findings have yet to be widely applied in clinical settings. We will examine the current barriers to implementing aortic hemodynamic models in clinical practice.

### 4.1 Aortic aneurysm

This review includes 17 studies on aortic aneurysms published between 2010 and 2024, detailed in the Supplementary Table S2 online. Of these, 14 studies focused on abdominal aortic aneurysms (AAA) and three on thoracic aortic aneurysm. The primary objective of these studies was to investigate the relationship between aneurysm volume expansion (Joly et al., 2020; Meyrignac et al., 2020; Bappoo et al., 2021; Rezaeitaleshmahalleh et al., 2024), progression (Arzani et al., 2014; Zambrano et al., 2016; McClarty et al., 2022; Salmasi et al., 2023), rupture (Boyd et al., 2016; Chisci et al., 2018; Qiu et al., 2019; 2022; Zhou et al., 2021; Teng et al., 2022), and hemodynamic characteristics. Additionally, the effects of physiological factors such as exercise (Les et al., 2010; Suh et al., 2011) and hypertension (Ramaekers et al., 2024) on these hemodynamic features were explored.

The methodologies employed in these studies included parallel control (12 studies) and longitudinal analysis (5 studies), either alone or in combination. The most common imaging technique was CTA (10 studies), followed by MRA (4 studies). Two studies used 4D-flow MRI, which allowed for the simultaneous acquisition of patient-specific inlet velocity profiles. One study adopted CECT for AAA imaging. Most studies modeled blood flow as a Newtonian fluid (12 studies) and assumed laminar flow (7 studies), with 14 studies conducting transient simulations. Regarding the boundary conditions, only one study modeled the vessel wall as a nonlinear, isotropic, hyperelastic material for FSI analysis (Teng et al., 2022), while the rest used a rigid, no-slip wall. For inlet boundary conditions, six studies used patient-specific, clinically measured flow rates, while the others relied on empirical values. At the outlets, nine studies applied three-element Windkessel models, while the remainder utilized empirical pressure values (5 studies) or flow distributions (1 study). In the postprocessing of hemodynamics, the most commonly assessed metrics were WSS, TAWSS, OSI, RRT, and endothelial cell activation potential (ECAP).

Three studies on aneurysm volume expansion confirmed a strong correlation between low baseline WSS and AAA expansion (Joly et al., 2020; Meyrignac et al., 2020; Bappoo et al., 2021). Furthermore, Joly et al. (2020) found that lower minimum TAWSS, along with higher RRT and ECAP values, were linked to a higher risk of rapid AAA expansion.

Two studies on intraluminal thrombus (ILT) progression in AAA yielded conflicting results. Arzani et al. (2014) found that low OSI was the primary factor associated with ILT growth, while low TAWSS showed no significant role in thrombus deposition. In contrast, Zambrano et al. (2016) identified low WSS as a key driver of ILT accumulation. These differences may stem from varying thresholds for low WSS. Arzani et al. (2014) considered WSS below 0.1 Pa as low, while Zambrano et al. (2016) identified the lowest TAWSS in their study at around 0.4 Pa. Despite these discrepancies, both studies agreed that thrombus deposition primarily occurred in recirculation zones.

Two studies on ascending thoracic aortic aneurysm progression found that elevated WSS was associated with local biomechanical deterioration (McClarty et al., 2022; Salmasi et al., 2023). In addition, Salmasi et al. (2023) suggested that a large angle between the left ventricular outflow tract and ascending aorta may contribute to the abnormal elevation of WSS.

Five of the six studies on AAA rupture showed that low WSS could distinguish aneurysms at high risk of rupture or predict rupture locations (Boyd et al., 2016; Chisci et al., 2018; Qiu et al., 2019; Zhou et al., 2021; Teng et al., 2022). Although Qiu et al. (2022) did not find significant differences in WSS values between ruptured and unruptured aneurysms, they observed a larger area of low WSS in the ruptured group. They also identified that a non-physiological flow pattern, characterized by a helical main flow channel with vortices, strongly associated with rupture risk. In addition, ILT deposition, which generally occurs in areas of blood recirculation (Boyd et al., 2016), was linked to rupture risk. Studies suggested that thin-layered ILT might offer a protective effect, while thick-layered ILT was considered a risk factor for rupture (Qiu et al., 2019; Zhou et al., 2021).

### 4.2 Aortic dissection

Nine studies on aortic dissections published between 2013 and 2023 were included, as detailed in the Supplementary Table S3 online. Of these, six studies were on TBAD and three on type A aortic dissection (TAAD). These studies mainly focused on the relationship between tear occurrence and hemodynamic characteristics (Chi et al., 2017; Hohri et al., 2021; Marrocco-Trischitta and Sturla, 2022; Wen et al., 2022; Wen et al., 2023; Williams et al., 2022; Li et al., 2023), as well as the correlation between hemodynamics and disease progression (Cheng et al., 2013; Shang et al., 2015).

All studies used parallel control methodology, with three incorporating longitudinal analysis and three combined virtual repairs. Seven studies used CTA imaging, while two studies adopted CECT for TAAD imaging. All studies performed transient simulations. In six studies, blood was modeled as a Newtonian fluid, whereas turbulence models were employed in three studies, and laminar flow was assumed in two others. For boundary conditions, all studies simplified the vessel wall as rigid and no-slip. Empirical flow waveforms were specified at inlet in all cases. At the outlet, four studies used three-element Windkessel models, while the rest utilized empirical pressure values. In the postprocessing of hemodynamic characteristics, the most commonly assessed metrics were WSS, TAWSS, OSI, and local normalized helicity (LNH).

Four studies on TBAD occurrence found a correlation between abnormal flow patterns, disturbed WSS, and tear location (Marrocco-Trischitta and Sturla, 2022; Wen et al., 2022; Wen et al., 2023; Li et al., 2023). These studies suggested that disrupted helical flow in the proximal descending aorta contributed to the initial tear formation. Additionally, metrics of WSS disturbance, such as transWSS (Li et al., 2023), cross-flow index (CFI) (Wen et al., 2022; Wen et al., 2023), and OSI (Wen et al., 2023), were significantly elevated in TBAD patients compared to healthy individuals. In TAAD, two studies linked elevated maximum WSS in the ascending aorta to an increased dissection risk (Chi et al., 2017; Williams et al., 2022). However, Hohri et al. (2021) found high OSI, rather than high WSS to be more closely associated with the tear location.

Two studies on TBAD progression suggested that higher flow rates in the false lumen were associated with both short- and long-term adverse outcomes, including malperfusion and rapid aneurysmal dilation (Cheng et al., 2013; Shang et al., 2015). Cheng et al. (2013) found that the flow rate in the false lumen correlated with the size and location of the primary entry tear, while Shang et al. (2015) observed a significant link between elevated TAWSS and aneurysmal dilation.

### 4.3 Limitations

Obviously, the studies included a limited number of patients, with a total of 1,047 subjects across 26 studies, the largest of which involved 295 subjects (Bappoo et al., 2021). Notably, 80% of the studies had fewer than 50 patients. Additionally, due to ethical concerns, it is difficult to track the severe progression of aortic aneurysms or dissections, which results in smaller patient cohorts for these conditions, making it harder to fully understand the role of hemodynamics. As an example, we can refer to the noninvasive fractional flow reserve, a hemodynamic indicator that has been used clinically to diagnose coronary stenosis. Prospective studies on this indicator, like the DISCOVER-FLOW study (Koo et al., 2011) and the DeFACTO study (Nakazato et al., 2013), involved over 100 patients and more than 400 vessels. However, given that the aorta is far more complex than the coronary arteries, larger patient cohorts will be necessary to establish a clear relationship between hemodynamics and disease progression in aortic conditions.

Additionally, many studies did not provide comprehensive descriptions of the parameters used in their CFD models, as summarized in Supplementary Tables S1, S2. Only five studies fully detailed their settings (Cheng et al., 2013; Qiu et al., 2019; Joly et al., 2020; Ramaekers et al., 2024; Rezaeitaleshmahalleh et al., 2024). Although this does not necessarily undermine the reliability of these studies, it does limit the applicability of their findings to other cohorts.

Moreover, patient-specific boundary conditions were seldom used, with only six studies employing clinically measured inlet flow waveforms. Although the use of empirical boundary conditions may compromise the accuracy of CFD models in reflecting true aortic hemodynamics, such simplifications can still be acceptable if they do not affect disease stratification, as demonstrated in studies on intracranial aneurysms (Li et al., 2020).

Finally, advanced modelling techniques were rarely employed in these large sample studies, likely due to the challenges of implementation and the substantial computational time required. For instance, most studies assumed a rigid, no-slip vessel wall, with only one study using a compliant wall for FSI simulations (Teng et al., 2022). However, rigid wall models fail to capture certain clinical features such as dynamic obstruction caused by intimal flap motion in aortic dissection (Kim et al., 2023). This highlights the need for further development and application of advanced modelling approaches in diverse clinical scenarios, focusing on reducing implementation complexity and improving overall performance.

## 5 Future directions

Despite the challenges associated with CFD modelling, such as complex computational processes and high computational demands, it shows considerable promise in the clinical management of cardiovascular diseases. However, significant uncertainties remain in accurately predicting the hemodynamics in aortic aneurysms and dissections. While existing studies have provided guidance on parameter settings for specific stages of CFD modelling, these insights remain insufficient for the full modelling process. Future research should focus on assessing the impact of different parameter settings on risk stratification through large-scale studies and provide well-founded recommendations.

Several large sample studies have demonstrated consistent associations between hemodynamics and aortic diseases, highlighting the potential of CFD-based hemodynamic analysis in clinical management. However, these studies are often constrained by limited sample sizes and insufficient detail on CFD parameters, such as turbulence model and convergence criteria. To address these limitations, future research should prioritize expanding cohort sizes and establishing standardized reporting protocols for computational modelling details.

This review outlines the workflow in CFD modelling for aortic hemodynamics and highlights recent advancements in the field. It also examines large sample studies to explore the relationship between hemodynamic features and aortic aneurysms and dissections. Our hope is to contribute to advancing the clinical application of CFD-based hemodynamic analysis in the management of these conditions.

## Glossary

CFD: computational fluid dynamics
TBAD: type B aortic dissection
DSA: digital subtraction angiography
CECT: contrast-enhanced computed tomography
CTA: computed tomography angiography
MRI: magnetic resonance imaging
MRA: magnetic resonance angiography
3DRA: 3D rotational angiography
IA: intracranial aneurysm
FSI: fluid-structure interaction
Re: Reynolds number
α: Womersley number
Rê: peak Reynolds number
DNS: direct numerical simulation
LES: large eddy simulation
SST: shear stress transport
WSS: wall shear stress
OSI: oscillatory shear index
GON: gradient oscillatory number
RRT: relative residence time
TAWSS: time-averaged wall shear stress
transWSS: transverse wall shear stress
AAA: abdominal aortic aneurysm
ECAP: endothelial cell activation potential
ILT: intraluminal thrombus
TAAD: type A aortic dissection
LNH: local normalized helicity
CFI: cross-flow index

## References

[B1] Abdelkhalek H. Abdelhameed E. A. Zakarea A. El Malky I. (2022). Predictors of flow diverter stent in large and giant unruptured intracranial aneurysms, single-center experience. Neurol. Sci. 43, 6399–6405. 10.1007/s10072-022-06336-w 35984605 PMC9616764

[B2] Algabri Y. A. Rookkapan S. Gramigna V. Espino D. M. Chatpun S. (2019). Computational study on hemodynamic changes in patient-specific proximal neck angulation of abdominal aortic aneurysm with time-varying velocity. Australas. Phys. Eng. Sci. Med. 42, 181–190. 10.1007/s13246-019-00728-7 30762222

[B3] Alimohammadi M. Sherwood J. M. Karimpour M. Agu O. Balabani S. Díaz-Zuccarini V. (2015). Aortic dissection simulation models for clinical support: fluid-structure interaction vs. rigid wall models. Biomed. Eng. OnLine 14, 34. 10.1186/s12938-015-0032-6 25881252 PMC4407424

[B4] Antiga L. Piccinelli M. Botti L. Ene-Iordache B. Remuzzi A. Steinman D. A. (2008). An image-based modeling framework for patient-specific computational hemodynamics. Med. Biol. Eng. Comput. 46, 1097–1112. 10.1007/s11517-008-0420-1 19002516

[B5] Armour C. H. Guo B. Pirola S. Saitta S. Liu Y. Dong Z. (2021). The influence of inlet velocity profile on predicted flow in type B aortic dissection. Biomech. Model. Mechanobiol. 20, 481–490. 10.1007/s10237-020-01395-4 33068193 PMC7979630

[B6] Arthurs C. J. Khlebnikov R. Melville A. Marčan M. Gomez A. Dillon-Murphy D. (2021). CRIMSON: an open-source software framework for cardiovascular integrated modelling and simulation. PLOS Comput. Biol. 17, e1008881. 10.1371/journal.pcbi.1008881 33970900 PMC8148362

[B7] Arzani A. Suh G.-Y. Dalman R. L. Shadden S. C. (2014). A longitudinal comparison of hemodynamics and intraluminal thrombus deposition in abdominal aortic aneurysms. Am. J. Physiol.-Heart Circ. Physiol. 307, H1786–H1795. 10.1152/ajpheart.00461.2014 25326533 PMC4269702

[B8] Ashkezari S. F. S. Mut F. Slawski M. Jimenez C. M. Robertson A. M. Cebral J. R. (2022). Identification of small, regularly shaped cerebral aneurysms prone to rupture. Am. J. Neuroradiol. 43, 547–553. 10.3174/ajnr.A7470 35332023 PMC8993208

[B9] Athani A. Ghazali N. N. N. Badruddin I. A. Kamangar S. Anqi A. E. Algahtani A. (2022). Investigation of two-way fluid-structure interaction of blood flow in a patient-specific left coronary artery. Biomed. Mater. Eng. 33, 13–30. 10.3233/BME-201171 34366314

[B10] Athani A. Ghazali N. N. N. Badruddin I. A. Usmani A. Y. Amir M. Singh D. (2024). Image-based hemodynamic and rheological study of patient’s diseased arterial vasculatures using computational fluid dynamics (CFD) and fluid–structure interactions (FSI) analysis: a review. Arch. Comput. Methods Eng. 10.1007/s11831-024-10193-5

[B11] Aycan O. Topuz A. Kadem L. (2023). Evaluating uncertainties in CFD simulations of patient-specific aorta models using Grid Convergence Index method. Mech. Res. Commun. 133, 104188. 10.1016/j.mechrescom.2023.104188

[B12] Azizi A. (2023). Applied complex flow: applications of complex flows and CFD. Singapore: Springer Nature Singapore. 10.1007/978-981-19-7746-6

[B13] Bappoo N. Syed M. B. J. Khinsoe G. Kelsey L. J. Forsythe R. O. Powell J. T. (2021). Low shear stress at baseline predicts expansion and aneurysm-related events in patients with abdominal aortic aneurysm. Circ. Cardiovasc. Imaging 14, 1112–1121. 10.1161/CIRCIMAGING.121.013160 34875845

[B14] Black S. M. Maclean C. Hall Barrientos P. Ritos K. McQueen A. Kazakidi A. (2023). Calibration of patient-specific boundary conditions for coupled CFD models of the aorta derived from 4D Flow-MRI. Front. Bioeng. Biotechnol. 11, 1178483. 10.3389/fbioe.2023.1178483 37251565 PMC10210162

[B15] Boccadifuoco A. Mariotti A. Capellini K. Celi S. Salvetti M. V. (2018). Validation of numerical simulations of thoracic aorta hemodynamics: comparison with *in vivo* measurements and stochastic sensitivity analysis. Cardiovasc. Eng. Technol. 9, 688–706. 10.1007/s13239-018-00387-x 30357714

[B16] Bonfanti M. Balabani S. Alimohammadi M. Agu O. Homer-Vanniasinkam S. Díaz-Zuccarini V. (2018). A simplified method to account for wall motion in patient-specific blood flow simulations of aortic dissection: comparison with fluid-structure interaction. Med. Eng. Phys. 58, 72–79. 10.1016/j.medengphy.2018.04.014 29759947

[B17] Bonfanti M. Balabani S. Greenwood J. P. Puppala S. Homer-Vanniasinkam S. Díaz-Zuccarini V. (2017). Computational tools for clinical support: a multi-scale compliant model for haemodynamic simulations in an aortic dissection based on multi-modal imaging data. J. R. Soc. Interface 14, 20170632. 10.1098/rsif.2017.0632 29118115 PMC5721167

[B18] Bonfanti M. Franzetti G. Homer-Vanniasinkam S. Díaz-Zuccarini V. Balabani S. (2020). A combined *in vivo*, *in vitro*, *in silico* approach for patient-specific haemodynamic studies of aortic dissection. Ann. Biomed. Eng. 48, 2950–2964. 10.1007/s10439-020-02603-z 32929558 PMC7723947

[B19] Bonfanti M. Franzetti G. Maritati G. Homer-Vanniasinkam S. Balabani S. Díaz-Zuccarini V. (2019). Patient-specific haemodynamic simulations of complex aortic dissections informed by commonly available clinical datasets. Med. Eng. Phys. 71, 45–55. 10.1016/j.medengphy.2019.06.012 31257054

[B20] Botar C. C. Tóth Á. Á. Klisurić O. R. Nićiforović D. D. Vučaj Ćirilović V. A. Till V. E. (2017). Dynamic simulation and Doppler Ultrasonography validation of blood flow behavior in Abdominal Aortic Aneurysm. Phys. Medica PM Int. J. Devoted Appl. Phys. Med. Biol. Off. J. Ital. Assoc. Biomed. Phys. AIFB 37, 1–8. 10.1016/j.ejmp.2017.03.021 28535909

[B21] Boyd A. J. Kuhn D. C. S. Lozowy R. J. Kulbisky G. P. (2016). Low wall shear stress predominates at sites of abdominal aortic aneurysm rupture. J. Vasc. Surg. 63, 1613–1619. 10.1016/j.jvs.2015.01.040 25752691

[B22] Caballero A. D. Laín S. (2013). A review on computational fluid dynamics modelling in human thoracic aorta. Cardiovasc. Eng. Technol. 4, 103–130. 10.1007/s13239-013-0146-6

[B23] Caballero A. D. Laín S. (2015). Numerical simulation of non-Newtonian blood flow dynamics in human thoracic aorta. Comput. Methods Biomech. Biomed. Engin. 18, 1200–1216. 10.1080/10255842.2014.887698 24559110

[B24] Calò K. Capellini K. De Nisco G. Mazzi V. Gasparotti E. Gallo D. (2023). Impact of wall displacements on the large-scale flow coherence in ascending aorta. J. Biomech. 154, 111620. 10.1016/j.jbiomech.2023.111620 37178494

[B25] Cao L. Shi R. Ge Y. Xing L. Zuo P. Jia Y. (2019). Fully automatic segmentation of type B aortic dissection from CTA images enabled by deep learning. Eur. J. Radiol. 121, 108713. 10.1016/j.ejrad.2019.108713 31683252

[B26] Capellini K. Gasparotti E. Cella U. Costa E. Fanni B. M. Groth C. (2021). A novel formulation for the study of the ascending aortic fluid dynamics with *in vivo* data. Med. Eng. Phys. 91, 68–78. 10.1016/j.medengphy.2020.09.005 33008714

[B27] Capellini K. Vignali E. Costa E. Gasparotti E. Biancolini M. E. Landini L. (2018). Computational fluid dynamic study for aTAA hemodynamics: an integrated image-based and radial basis functions mesh morphing approach. J. Biomech. Eng. 140, 111007. 10.1115/1.4040940 30098137

[B28] Cebral J. R. Meng H. (2012). Counterpoint: realizing the clinical utility of computational fluid dynamics—closing the gap. Am. J. Neuroradiol. 33, 396–398. 10.3174/ajnr.A2994 22282452 PMC7966449

[B29] Chandran K. B. (1993). Flow dynamics in the human aorta. J. Biomech. Eng. 115, 611–616. 10.1115/1.2895548 8302049

[B30] Chen D. Zhang X. Mei Y. Liao F. Xu H. Li Z. (2021). Multi-stage learning for segmentation of aortic dissections using a prior aortic anatomy simplification. Med. Image Anal. 69, 101931. 10.1016/j.media.2020.101931 33618153

[B31] Cheng Z. Juli C. Wood N. B. Gibbs R. G. J. Xu X. Y. (2014). Predicting flow in aortic dissection: comparison of computational model with PC-MRI velocity measurements. Med. Eng. Phys. 36, 1176–1184. 10.1016/j.medengphy.2014.07.006 25070022

[B32] Cheng Z. Riga C. Chan J. Hamady M. Wood N. B. Cheshire N. J. W. (2013). Initial findings and potential applicability of computational simulation of the aorta in acute type B dissection. J. Vasc. Surg. 57, 35S–43S. 10.1016/j.jvs.2012.07.061 23336853

[B33] Cheng Z. Wood N. B. Gibbs R. G. J. Xu X. Y. (2015). Geometric and flow features of type B aortic dissection: initial findings and comparison of medically treated and stented cases. Ann. Biomed. Eng. 43, 177–189. 10.1007/s10439-014-1075-8 25092420

[B34] Cherry M. Khatir Z. Khan A. Bissell M. (2022). The impact of 4D-Flow MRI spatial resolution on patient-specific CFD simulations of the thoracic aorta. Sci. Rep. 12, 15128–15216. 10.1038/s41598-022-19347-6 36068322 PMC9448751

[B35] Chi Q. Chen H. Yang S. Mu L. Ji C. He Y. (2022a). Study of effect of boundary conditions on patient-specific aortic hemodynamics. Comput. Model. Eng. Sci. 131, 31–47. 10.32604/cmes.2022.018286

[B36] Chi Q. He Y. Luan Y. Qin K. Mu L. (2017). Numerical analysis of wall shear stress in ascending aorta before tearing in type A aortic dissection. Comput. Biol. Med. 89, 236–247. 10.1016/j.compbiomed.2017.07.029 28843154

[B37] Chi Z. Beile L. Deyu L. Yubo F. (2022b). Application of multiscale coupling models in the numerical study of circulation system. Med. Nov. Technol. Devices 14, 100117. 10.1016/j.medntd.2022.100117

[B38] Chisci E. Alamanni N. Iacoponi F. Michelagnoli S. Procacci T. Colombo G. (2018). Grading abdominal aortic aneurysm rupture risk. J. Cardiovasc. Surg. (Torino) 59, 87–94. 10.23736/S0021-9509.16.08848-0 26184567

[B39] Chnafa C. Brina O. Pereira V. M. Steinman D. A. (2018). Better than nothing: a rational approach for minimizing the impact of outflow strategy on cerebrovascular simulations. AJNR Am. J. Neuroradiol. 39, 337–343. 10.3174/ajnr.A5484 29269407 PMC7410584

[B40] Dabagh M. Vasava P. Jalali P. (2015). Effects of severity and location of stenosis on the hemodynamics in human aorta and its branches. Med. Biol. Eng. Comput. 53, 463–476. 10.1007/s11517-015-1253-3 25725629

[B41] Dai Y. Luo G. Dai X. Liu H. (2020). Hemodynamic effects of multiple overlapping uncovered stents on aortic dissection: surgical strategies and implications for false lumen thrombosis. Cardiovasc. Eng. Technol. 11, 24–35. 10.1007/s13239-019-00443-0 31820352

[B42] Dennis K. D. Kallmes D. F. Dragomir-Daescu D. (2017). Cerebral aneurysm blood flow simulations are sensitive to basic solver settings. J. Biomech. 57, 46–53. 10.1016/j.jbiomech.2017.03.020 28395878

[B43] Ding Z. Liu Q. Luo H. Yang M. Zhang Y. Wang S. (2024). A preoperative planning procedure of septal myectomy for hypertrophic obstructive cardiomyopathy using image-based computational fluid dynamics simulations and shape optimization. Sci. Rep. 14, 24617. 10.1038/s41598-024-74091-3 39426997 PMC11490630

[B44] Ene F. Delassus P. Morris L. (2014). The influence of computational assumptions on analysing abdominal aortic aneurysm haemodynamics. Proc. Inst. Mech. Eng. H. 228, 768–780. 10.1177/0954411914546122 25085698

[B45] Etli M. Canbolat G. Karahan O. Koru M. (2021). Numerical investigation of patient-specific thoracic aortic aneurysms and comparison with normal subject via computational fluid dynamics (CFD). Med. Biol. Eng. Comput. 59, 71–84. 10.1007/s11517-020-02287-6 33225424

[B46] Evangelista A. Isselbacher E. M. Bossone E. Gleason T. G. Eusanio M. D. Sechtem U. (2018). Insights from the international registry of acute aortic dissection. Circulation 137, 1846–1860. 10.1161/CIRCULATIONAHA.117.031264 29685932

[B47] Evju Ø. Pozo J. M. Frangi A. F. Mardal K.-A. (2017). Robustness of common hemodynamic indicators with respect to numerical resolution in 38 middle cerebral artery aneurysms. PloS One 12, e0177566. 10.1371/journal.pone.0177566 28609457 PMC5469453

[B48] Feiger B. Gounley J. Adler D. Leopold J. A. Draeger E. W. Chaudhury R. (2020). Accelerating massively parallel hemodynamic models of coarctation of the aorta using neural networks. Sci. Rep. 10, 9508. 10.1038/s41598-020-66225-0 32528104 PMC7289812

[B49] Filipovic N. Nikolic D. Saveljic I. Djukic T. Adjic O. Kovacevic P. (2013). Computer simulation of thromboexclusion of the complete aorta in the treatment of chronic type B aneurysm. Comput. Aided Surg. 18, 1–9. 10.3109/10929088.2012.741145 23176116

[B50] François C. J. Tuite D. Deshpande V. Jerecic R. Weale P. Carr J. C. (2008). Unenhanced MR angiography of the thoracic aorta: initial clinical evaluation. Am. J. Roentgenol. 190, 902–906. 10.2214/AJR.07.2997 18356435

[B51] Gao J. Cao H. Hu G. Wu Y. Xu Y. Cui H. (2023). The mechanism and therapy of aortic aneurysms. Signal Transduct. Target. Ther. 8, 55–20. 10.1038/s41392-023-01325-7 36737432 PMC9898314

[B52] Garber L. Khodaei S. Keshavarz-Motamed Z. (2022). The critical role of lumped parameter models in patient-specific cardiovascular simulations. Arch. Comput. Methods Eng. 29, 2977–3000. 10.1007/s11831-021-09685-5

[B53] Geiger J. Hirtler D. Gottfried K. Rahman O. Bollache E. Barker A. J. (2016). Longitudinal evaluation of aortic hemodynamics in marfan syndrome: new insights from a 4D flow cardiovascular magnetic resonance multi-year follow-up study. J. Cardiovasc. Magn. Reson. 19, 33. 10.1186/s12968-017-0347-5 PMC5361800 28327193

[B54] Hall J. E. Guyton A. C. (2011). Guyton and Hall textbook of medical physiology. 12th ed. Philadelphia, Pa: Saunders/Elsevier.

[B55] Hirschhorn M. Tchantchaleishvili V. Stevens R. Rossano J. Throckmorton A. (2020). Fluid–structure interaction modeling in cardiovascular medicine – a systematic review 2017–2019. Med. Eng. Phys. 78, 1–13. 10.1016/j.medengphy.2020.01.008 32081559

[B56] Hodis S. Uthamaraj S. Smith A. L. Dennis K. D. Kallmes D. F. Dragomir-Daescu D. (2012). Grid convergence errors in hemodynamic solution of patient-specific cerebral aneurysms. J. Biomech. 45, 2907–2913. 10.1016/j.jbiomech.2012.07.030 23062796

[B57] Hohri Y. Numata S. Itatani K. Kanda K. Yamazaki S. Inoue T. (2021). Prediction for future occurrence of type A aortic dissection using computational fluid dynamics. Eur. J. Cardio-Thorac. Surg. Off. J. Eur. Assoc. Cardio-Thorac. Surg. 60, 384–391. 10.1093/ejcts/ezab094 33619516

[B58] Hu L. Wang Y. Rao J. Tan L. He M. Zeng X. (2024). Computed tomography-derived fractional flow reserve: developing A gold standard for coronary artery disease diagnostics. Rev. Cardiovasc. Med. 25, 372. 10.31083/j.rcm2510372 39484113 PMC11522765

[B59] Hu M. Zhang Z. Liu M. (2023). Transient particle transport prediction based on lattice Boltzmann method-based large eddy simulation and Markov chain model. Build. Simul. 16, 1135–1148. 10.1007/s12273-023-0995-3

[B60] Hua Y. Oh J. H. Kim Y. B. (2015). Influence of parent artery segmentation and boundary conditions on hemodynamic characteristics of intracranial aneurysms. Yonsei Med. J. 56, 1328–1337. 10.3349/ymj.2015.56.5.1328 26256976 PMC4541663

[B61] Humphrey J. D. Holzapfel G. A. (2012). Mechanics, mechanobiology, and modeling of human abdominal aorta and aneurysms. J. Biomech. 45, 805–814. 10.1016/j.jbiomech.2011.11.021 22189249 PMC3294195

[B62] Jayendiran R. Nour B. Ruimi A. (2018). Computational fluid–structure interaction analysis of blood flow on patient-specific reconstructed aortic anatomy and aneurysm treatment with Dacron graft. J. Fluids Struct. 81, 693–711. 10.1016/j.jfluidstructs.2018.06.008

[B63] Joly F. Soulez G. Lessard S. Kauffmann C. Vignon-Clementel I. (2020). A cohort longitudinal study identifies morphology and hemodynamics predictors of abdominal aortic aneurysm growth. Ann. Biomed. Eng. 48, 606–623. 10.1007/s10439-019-02375-1 31637578

[B64] Kamada H. Nakamura M. Ota H. Higuchi S. Takase K. (2022). Blood flow analysis with computational fluid dynamics and 4D-flow MRI for vascular diseases. J. Cardiol. 80, 386–396. 10.1016/j.jjcc.2022.05.007 35718672

[B65] Kannojiya V. Das A. K. Das P. K. (2021). Simulation of blood as fluid: a review from rheological aspects. IEEE Rev. Biomed. Eng. 14, 327–341. 10.1109/RBME.2020.3011182 32746370

[B66] Katz S. Caiazzo A. Moreau B. Wilbrandt U. Brüning J. Goubergrits L. (2023). Impact of turbulence modeling on the simulation of blood flow in aortic coarctation. Int. J. Numer. Methods Biomed. Eng. 39, e3695. 10.1002/cnm.3695 36914373

[B67] Kim T. van Bakel P. A. J. Nama N. Burris N. Patel H. J. Williams D. M. (2023). A computational study of dynamic obstruction in type B aortic dissection. J. Biomech. Eng. 145, 031008. 10.1115/1.4056355 36459144 PMC10854260

[B68] Koo B.-K. Erglis A. Doh J.-H. Daniels D. V. Jegere S. Kim H.-S. (2011). Diagnosis of ischemia-causing coronary stenoses by noninvasive fractional flow reserve computed from coronary computed tomographic angiograms. J. Am. Coll. Cardiol. 58, 1989–1997. 10.1016/j.jacc.2011.06.066 22032711

[B69] Kousera C. A. Wood N. B. Seed W. A. Torii R. O’Regan D. Xu X. Y. (2013). A numerical study of aortic flow stability and comparison with *in vivo* flow measurements. J. Biomech. Eng. 135, 011003. 10.1115/1.4023132 23363214

[B70] Larrabide I. Omedas P. Martelli Y. Planes X. Nieber M. Moya J. A. (2009). “GIMIAS: an open source framework for efficient development of research tools and clinical prototypes,” in Functional imaging and modeling of the heart. Editors Ayache N. Delingette H. Sermesant M. (Berlin, Heidelberg: Springer), 417–426. 10.1007/978-3-642-01932-6_45

[B71] Lassila T. Sarrami-Foroushani A. Hejazi S. Frangi A. F. (2020). Population-specific modelling of between/within-subject flow variability in the carotid arteries of the elderly. Int. J. Numer. Methods Biomed. Eng. 36, e3271. 10.1002/cnm.3271 31691518

[B72] Lee S.-W. Antiga L. Steinman D. A. (2009). Correlations among indicators of disturbed flow at the normal carotid bifurcation. J. Biomech. Eng. 131, 061013. 10.1115/1.3127252 19449967

[B73] LeMaire S. A. Russell L. (2011). Epidemiology of thoracic aortic dissection. Nat. Rev. Cardiol. 8, 103–113. 10.1038/nrcardio.2010.187 21173794

[B74] Les A. S. Shadden S. C. Figueroa C. A. Park J. M. Tedesco M. M. Herfkens R. J. (2010). Quantification of hemodynamics in abdominal aortic aneurysms during rest and exercise using magnetic resonance imaging and computational fluid dynamics. Ann. Biomed. Eng. 38, 1288–1313. 10.1007/s10439-010-9949-x 20143263 PMC6203348

[B75] Lesage D. Angelini E. D. Bloch I. Funka-Lea G. (2009). A review of 3D vessel lumen segmentation techniques: models, features and extraction schemes. Med. Image Anal. 13, 819–845. 10.1016/j.media.2009.07.011 19818675

[B76] Li D. Wang J. Zeng W. Zeng X. Liu Z. Cao H. (2023). The loss of helical flow in the thoracic aorta might be an identifying marker for the risk of acute type B aortic dissection. Comput. Methods Programs Biomed. 230, 107331. 10.1016/j.cmpb.2022.107331 36621070

[B77] Li W. Wang S. Tian Z. Zhu W. Zhang Y. Zhang Y. (2020). Discrimination of intracranial aneurysm rupture status: patient-specific inflow boundary may not be a must-have condition in hemodynamic simulations. Neuroradiology 62, 1485–1495. 10.1007/s00234-020-02473-1 32588092

[B78] Liu Y. Jiang G. Wang X. An X. Wang F. (2023). The relationship between geometry and hemodynamics of the stenotic carotid artery based on computational fluid dynamics. Clin. Neurol. Neurosurg. 231, 107860. 10.1016/j.clineuro.2023.107860 37390570

[B79] Liu Y. Li W. Ding Z. Tang Z. Luo Y. Hu J. (2025). Long-term longitudinal computational study of a marfan syndrome patient after hybrid repair of aortic arch dissection with parallel stent-grafts. Int. J. Numer. Methods Biomed. Eng. 41, e70018. 10.1002/cnm.70018 39979238

[B80] Long Ko J. K. Liu R. W. Ma D. Shi L. Ho Yu S. C. Wang D. (2017). Pulsatile hemodynamics in patient-specific thoracic aortic dissection models constructed from computed tomography angiography. J. X-Ray Sci. Technol. 25, 233–245. 10.3233/XST-17256 28234275

[B81] Maher G. Parker D. Wilson N. Marsden A. (2020). Neural network vessel lumen regression for automated lumen cross-section segmentation in cardiovascular image-based modeling. Cardiovasc. Eng. Technol. 11, 621–635. 10.1007/s13239-020-00497-5 33179176 PMC7785699

[B82] Maher G. D. Fleeter C. M. Schiavazzi D. E. Marsden A. L. (2021). Geometric uncertainty in patient-specific cardiovascular modeling with convolutional dropout networks. Comput. Methods Appl. Mech. Eng. 386, 114038. 10.1016/j.cma.2021.114038 34737480 PMC8562598

[B83] Mansouri H. Kemerli M. MacIver R. Amili O. (2024). Development of idealized human aortic models for *in vitro* and *in silico* hemodynamic studies. Front. Cardiovasc. Med. 11, 1358601. 10.3389/fcvm.2024.1358601 39161662 PMC11330894

[B84] Marrero V. L. Tichy J. A. Sahni O. Jansen K. E. (2014). Numerical study of purely viscous non-Newtonian flow in an abdominal aortic aneurysm. J. Biomech. Eng. 136, 101001. 10.1115/1.4027488 24769921

[B85] Marrocco-Trischitta M. M. Sturla F. (2022). Blood flow helical pattern in type III arch configuration as a potential risk factor for type B aortic dissection. Eur. J. Cardiothorac. Surg. 61, 132–139. 10.1093/ejcts/ezab307 34374753

[B86] Martínez A. Hoeijmakers M. Geronzi L. Morgenthaler V. Tomasi J. Rochette M. (2023). Effect of turbulence and viscosity models on wall shear stress derived biomarkers for aorta simulations. Comput. Biol. Med. 167, 107603. 10.1016/j.compbiomed.2023.107603 37922602

[B87] McClarty D. Ouzounian M. Tang M. Eliathamby D. Romero D. Nguyen E. (2022). Ascending aortic aneurysm haemodynamics are associated with aortic wall biomechanical properties. Eur. J. Cardio-Thorac. Surg. Off. J. Eur. Assoc. Cardio-Thorac. Surg. 61, 367–375. 10.1093/ejcts/ezab471 34718497

[B88] Meyrignac O. Bal L. Zadro C. Vavasseur A. Sewonu A. Gaudry M. (2020). Combining volumetric and wall shear stress analysis from CT to assess risk of abdominal aortic aneurysm progression. Radiology 295, 722–729. 10.1148/radiol.2020192112 32228297

[B89] Midulla M. Moreno R. Baali A. Chau M. Negre-Salvayre A. Nicoud F. (2012). Haemodynamic imaging of thoracic stent-grafts by computational fluid dynamics (CFD): presentation of a patient-specific method combining magnetic resonance imaging and numerical simulations. Eur. Radiol. 22, 2094–2102. 10.1007/s00330-012-2465-7 22645039

[B90] Mirzaee H. Henn T. Krause M. J. Goubergrits L. Schumann C. Neugebauer M. (2017). MRI-based computational hemodynamics in patients with aortic coarctation using the lattice Boltzmann methods: clinical validation study. J. Magn. Reson. Imaging JMRI 45, 139–146. 10.1002/jmri.25366 27384018 PMC5213689

[B91] Miyazaki S. Itatani K. Furusawa T. Nishino T. Sugiyama M. Takehara Y. (2017). Validation of numerical simulation methods in aortic arch using 4D Flow MRI. Heart Vessels 32, 1032–1044. 10.1007/s00380-017-0979-2 28444501 PMC5519664

[B92] Morab S. R. Sharma A. Murallidharan J. S. (2024). Computational hemodynamics in human vasculature: a review on role of rheology, multiphase flow, and fluid–structure interaction. J. Indian Inst. Sci. 104, 13–38. 10.1007/s41745-024-00425-9

[B93] Morbiducci U. Ponzini R. Gallo D. Bignardi C. Rizzo G. (2013). Inflow boundary conditions for image-based computational hemodynamics: impact of idealized versus measured velocity profiles in the human aorta. J. Biomech. 46, 102–109. 10.1016/j.jbiomech.2012.10.012 23159094

[B94] Moretti S. Tauro F. Orrico M. Mangialardi N. Facci A. L. (2023). Comparative analysis of patient-specific aortic dissections through computational fluid dynamics suggests increased likelihood of degeneration in partially thrombosed false lumen. Bioengineering 10, 316. 10.3390/bioengineering10030316 36978707 PMC10045026

[B169] Morris P. D. Narracott A. Von Tengg-Kobligk H. Silva Soto D. A. Hsiao S. Lungu A. (2016). Computational fluid dynamics modelling in cardiovascular medicine. Heart 102, 18–28. 10.1136/heartjnl-2015-308044 26512019 PMC4717410

[B95] Mustafa A. G. Allouh M. Z. Ghaida J. H. A. Al-Omari M. H. Mahmoud W. A. (2017). Branching patterns of the aortic arch: a computed tomography angiography-based study. Surg. Radiol. Anat. 39, 235–242. 10.1007/s00276-016-1720-z 27338939

[B96] Mutlu O. Salman H. E. Al-Thani H. El-Menyar A. Qidwai U. A. Yalcin H. C. (2023). How does hemodynamics affect rupture tissue mechanics in abdominal aortic aneurysm: focus on wall shear stress derived parameters, time-averaged wall shear stress, oscillatory shear index, endothelial cell activation potential, and relative residence time. Comput. Biol. Med. 154, 106609. 10.1016/j.compbiomed.2023.106609 36724610

[B97] Nakazato R. Park H.-B. Berman D. S. Gransar H. Koo B.-K. Erglis A. (2013). Noninvasive fractional flow reserve derived from computed tomography angiography for coronary lesions of intermediate stenosis severity. Circ. Cardiovasc. Imaging. 6, 881–889. 10.1161/CIRCIMAGING.113.000297 24081777

[B98] Nerem R. M. Seed W. A. Wood N. B. (1972). An experimental study of the velocity distribution and transition to turbulence in the aorta. J. Fluid Mech. 52, 137–160. 10.1017/S0022112072003003

[B99] Ong C. W. Kabinejadian F. Xiong F. Wong Y. R. Toma M. Nguyen Y. N. (2019). Pulsatile flow investigation in development of thoracic aortic aneurysm: an in-vitro validated fluid structure interaction analysis. J. Appl. Fluid Mech. 12, 1855–1872. 10.29252/jafm.12.06.29769

[B100] O’Rourke M. J. McCullough J. P. Kelly S. (2012). An investigation of the relationship between hemodynamics and thrombus deposition within patient-specific models of abdominal aortic aneurysm. Proc. Inst. Mech. Eng. H. 226, 548–564. 10.1177/0954411912444080 22913102

[B101] Pagoulatou S. Adamopoulos D. Rovas G. Bikia V. Stergiopulos N. (2021). Acute and long-term effects of aortic compliance decrease on central hemodynamics: a modeling analysis. Front. Physiol. 12, 701154. 10.3389/fphys.2021.701154 34381376 PMC8350396

[B102] Pant S. Fabrèges B. Gerbeau J. Vignon‐Clementel I. E. (2014). A methodological paradigm for patient‐specific multi‐scale CFD simulations: from clinical measurements to parameter estimates for individual analysis. Int. J. Numer. Methods Biomed. Eng. 30, 1614–1648. 10.1002/cnm.2692 25345820

[B103] Paritala P. K. Anbananthan H. Hautaniemi J. Smith M. George A. Allenby M. (2023). Reproducibility of the computational fluid dynamic analysis of a cerebral aneurysm monitored over a decade. Sci. Rep. 13, 219. 10.1038/s41598-022-27354-w 36604495 PMC9816094

[B104] Peiffer V. Sherwin S. J. Weinberg P. D. (2013). Does low and oscillatory wall shear stress correlate spatially with early atherosclerosis? A systematic review. Cardiovasc. Res. 99, 242–250. 10.1093/cvr/cvt044 23459102 PMC3695746

[B105] Peng L. Qiu Y. Yang Z. Yuan D. Dai C. Li D. (2019). Patient-specific computational hemodynamic analysis for interrupted aortic arch in an adult: implications for aortic dissection initiation. Sci. Rep. 9, 8600. 10.1038/s41598-019-45097-z 31197221 PMC6565632

[B106] Pereira V. M. Brina O. Marcos Gonzales A. Narata A. P. Bijlenga P. Schaller K. (2013). Evaluation of the influence of inlet boundary conditions on computational fluid dynamics for intracranial aneurysms: a virtual experiment. J. Biomech. 46, 1531–1539. 10.1016/j.jbiomech.2013.03.024 23602597

[B107] Perera R. Isoda H. Ishiguro K. Mizuno T. Takehara Y. Terada M. (2020). Assessing the risk of intracranial aneurysm rupture using morphological and hemodynamic biomarkers evaluated from magnetic resonance fluid dynamics and computational fluid dynamics. Magn. Reson. Med. Sci. MRMS Off. J. Jpn. Soc. Magn. Reson. Med. 19, 333–344. 10.2463/mrms.mp.2019-0107 PMC7809142 31956175

[B108] Pieper S. Halle M. Kikinis R. (2004). 3D slicer. 2004 2nd IEEE International Symposium on Biomedical Imaging: Nano to Macro (IEEE Cat No. 04EX821) 1, 632–635. 10.1109/ISBI.2004.1398617

[B109] Poelma C. Watton P. N. Ventikos Y. (2015). Transitional flow in aneurysms and the computation of haemodynamic parameters. J. R. Soc. Interface 12, 20141394. 10.1098/rsif.2014.1394 25694540 PMC4387528

[B110] Prakash S. Ethier C. R. (2001). Requirements for mesh resolution in 3D computational hemodynamics. J. Biomech. Eng. 123, 134–144. 10.1115/1.1351807 11340874

[B111] Qiao Y. Zeng Y. Ding Y. Fan J. Luo K. Zhu T. (2019). Numerical simulation of two-phase non-Newtonian blood flow with fluid-structure interaction in aortic dissection. Comput. Methods Biomech. Biomed. Engin. 22, 620–630. 10.1080/10255842.2019.1577398 30822150

[B112] Qiu Y. Wang J. Zhao J. Wang T. Zheng T. Yuan D. (2022). Association between blood flow pattern and rupture risk of abdominal aortic aneurysm based on computational fluid dynamics. Eur. J. Vasc. Endovasc. Surg. Off. J. Eur. Soc. Vasc. Surg. 64, 155–164. 10.1016/j.ejvs.2022.05.027 35605907

[B113] Qiu Y. Wang Y. Fan Y. Peng L. Liu R. Zhao J. (2019). Role of intraluminal thrombus in abdominal aortic aneurysm ruptures: a hemodynamic point of view. Med. Phys. 46, 4263–4275. 10.1002/mp.13658 31206182

[B114] Qiu Y. Yuan D. Wang Y. Wen J. Zheng T. (2018). Hemodynamic investigation of a patient-specific abdominal aortic aneurysm with iliac artery tortuosity. Comput. Methods Biomech. Biomed. Engin. 21, 824–833. 10.1080/10255842.2018.1522531 30398069

[B115] Ramaekers M. J. F. G. van der Vlugt I. B. Westenberg J. J. M. Perinajová R. Lamb H. J. Wildberger J. E. (2024). Flow patterns in ascending aortic aneurysms: determining the role of hypertension using phase contrast magnetic resonance and computational fluid dynamics. Comput. Biol. Med. 172, 108310. 10.1016/j.compbiomed.2024.108310 38508054

[B116] Ramaekers M. J. F. G. Westenberg J. J. M. Adriaans B. P. Nijssen E. C. Wildberger J. E. Lamb H. J. (2023). A clinician’s guide to understanding aortic 4D flow MRI. Insights Imaging 14, 114. 10.1186/s13244-023-01458-x 37395817 PMC10317921

[B117] Ramiro M. Franck N. Veunac L. Rousseau H. (2006). Non-linear transformation field to build moving meshes for patient specific blood flow simulations.

[B118] Rayz V. L. Boussel L. Acevedo-Bolton G. Martin A. J. Young W. L. Lawton M. T. (2008). Numerical simulations of flow in cerebral aneurysms: comparison of CFD results and *in vivo* MRI measurements. J. Biomech. Eng. 130, 051011. 10.1115/1.2970056 19045518

[B119] Rayz V. L. Cohen-Gadol A. A. (2020). Hemodynamics of cerebral aneurysms: connecting medical imaging and biomechanical analysis. Annu. Rev. Biomed. Eng. 22, 231–256. 10.1146/annurev-bioeng-092419-061429 32212833 PMC9219593

[B120] Ren Y. Chen G.-Z. Liu Z. Cai Y. Lu G.-M. Li Z.-Y. (2016). Reproducibility of image-based computational models of intracranial aneurysm: a comparison between 3D rotational angiography, CT angiography and MR angiography. Biomed. Eng. Online 15, 50. 10.1186/s12938-016-0163-4 27150439 PMC4858827

[B121] Rezaeitaleshmahalleh M. Lyu Z. Mu N. Wang M. Zhang X. Rasmussen T. E. (2024). Computational hemodynamics-based growth prediction for small abdominal aortic aneurysms: laminar simulations versus large eddy simulations. Ann. Biomed. Eng. 52, 3078–3097. 10.1007/s10439-024-03572-3 39020077 PMC11772086

[B122] Rosset A. Spadola L. Ratib O. (2004). OsiriX: an open-source software for navigating in multidimensional DICOM images. J. Digit. Imaging 17, 205–216. 10.1007/s10278-004-1014-6 15534753 PMC3046608

[B123] Salmasi M. Y. Pirola S. Mahuttanatan S. Fisichella S. M. Sengupta S. Jarral O. A. (2023). Geometry and flow in ascending aortic aneurysms are influenced by left ventricular outflow tract orientation: detecting increased wall shear stress on the outer curve of proximal aortic aneurysms. J. Thorac. Cardiovasc. Surg. 166, 11–21.e1. 10.1016/j.jtcvs.2021.06.014 34217540

[B124] Shang E. K. Nathan D. P. Fairman R. M. Bavaria J. E. Gorman R. C. Gorman J. H. (2015). Use of computational fluid dynamics studies in predicting aneurysmal degeneration of acute type B aortic dissections. J. Vasc. Surg. 62, 279–284. 10.1016/j.jvs.2015.02.048 25935270 PMC6685432

[B125] Shi Y. Lawford P. Hose R. (2011). Review of zero-D and 1-D models of blood flow in the cardiovascular system. Biomed. Eng. OnLine 10, 33. 10.1186/1475-925X-10-33 21521508 PMC3103466

[B126] Shimogonya Y. Ishikawa T. Imai Y. Matsuki N. Yamaguchi T. (2009). Can temporal fluctuation in spatial wall shear stress gradient initiate a cerebral aneurysm? A proposed novel hemodynamic index, the gradient oscillatory number (GON). J. Biomech. 42, 550–554. 10.1016/j.jbiomech.2008.10.006 19195658

[B127] Signorelli F. Gory B. Riva R. Labeyrie P. Pelissou-Guyotat I. Turjman F. (2015). Hemodynamics, inflammation, vascular remodeling, and the development and rupture of intracranial aneurysms: a review. Neuroimmunol. Neuroinflamm. 2, 59. 10.4103/2347-8659.154885

[B128] Signorelli F. Sela S. Gesualdo L. Chevrel S. Tollet F. Pailler-Mattei C. (2018). Hemodynamic stress, inflammation, and intracranial aneurysm development and rupture: a systematic review. World Neurosurg. 115, 234–244. 10.1016/j.wneu.2018.04.143 29709752

[B129] Soudah E. Rossi R. Idelsohn S. Oñate E. (2014). A reduced-order model based on the coupled 1D-3D finite element simulations for an efficient analysis of hemodynamics problems. Comput. Mech. 54, 1013–1022. 10.1007/s00466-014-1040-2

[B130] Spanos K. Eckstein H.-H. Giannoukas A. D. (2020). Small abdominal aortic aneurysms are not all the same. Angiology 71, 205–207. 10.1177/0003319719862965 31315421

[B131] Steinman D. A. (2002). Image-based computational fluid dynamics modeling in realistic arterial geometries. Ann. Biomed. Eng. 30, 483–497. 10.1114/1.1467679 12086000

[B132] Steinman D. A. Pereira V. M. (2019). How patient specific are patient-specific computational models of cerebral aneurysms? An overview of sources of error and variability. Neurosurg. Focus 47, E14. 10.3171/2019.4.FOCUS19123 31261118

[B133] Stokes C. Ahmed D. Lind N. Haupt F. Becker D. Hamilton J. (2023a). Aneurysmal growth in type-B aortic dissection: assessing the impact of patient-specific inlet conditions on key haemodynamic indices. J. R. Soc. Interface 20, 20230281. 10.1098/rsif.2023.0281 37727072 PMC10509589

[B134] Stokes C. Bonfanti M. Li Z. Xiong J. Chen D. Balabani S. (2021). A novel MRI-based data fusion methodology for efficient, personalised, compliant simulations of aortic haemodynamics. J. Biomech. 129, 110793. 10.1016/j.jbiomech.2021.110793 34715606 PMC8907869

[B135] Stokes C. Haupt F. Becker D. Muthurangu V. von Tengg-Kobligk H. Balabani S. (2023b). The influence of minor aortic branches in patient-specific flow simulations of type-B aortic dissection. Ann. Biomed. Eng. 51, 1627–1644. 10.1007/s10439-023-03175-4 36967447 PMC10264290

[B136] Suh G.-Y. Les A. S. Tenforde A. S. Shadden S. C. Spilker R. L. Yeung J. J. (2011). Hemodynamic changes quantified in abdominal aortic aneurysms with increasing exercise intensity using mr exercise imaging and image-based computational fluid dynamics. Ann. Biomed. Eng. 39, 2186–2202. 10.1007/s10439-011-0313-6 21509633 PMC3362397

[B137] Sveinsson Cepero N. Shadden S. C. (2025). SeqSeg: learning local segments for automatic vascular model construction. Ann. Biomed. Eng. 53, 158–179. 10.1007/s10439-024-03611-z 39292327 PMC11782360

[B138] Tan F. P. P. Torii R. Borghi A. Mohiaddin R. H. Wood N. B. Xu X. Y. (2009). Fluid-structure interaction analysis of wall stress and flow patterns in a thoracic aortic aneurysm. Int. J. Appl. Mech. 01, 179–199. 10.1142/S1758825109000095

[B139] Tanweer O. Wilson T. A. Metaxa E. Riina H. A. Meng H. (2014). A comparative review of the hemodynamics and pathogenesis of cerebral and abdominal aortic aneurysms: lessons to learn from each other. J. Cerebrovasc. Endovasc. Neurosurg. 16, 335–349. 10.7461/jcen.2014.16.4.335 25599042 PMC4296046

[B140] Teng B. Zhou Z. Zhao Y. Wang Z. (2022). Combined curvature and wall shear stress analysis of abdominal aortic aneurysm: an analysis of rupture risk factors. Cardiovasc. Interv. Radiol. 45, 752–760. 10.1007/s00270-022-03140-z PMC9117347 35415808

[B141] Tse K. M. Chang R. Lee H. P. Lim S. P. Venkatesh S. K. Ho P. (2013). A computational fluid dynamics study on geometrical influence of the aorta on haemodynamics. Eur. J. Cardio-Thorac. Surg. Off. J. Eur. Assoc. Cardio-Thorac. Surg. 43, 829–838. 10.1093/ejcts/ezs388 22766960

[B142] Updegrove A. Wilson N. M. Merkow J. Lan H. Marsden A. L. Shadden S. C. (2017). SimVascular: an open source pipeline for cardiovascular simulation. Ann. Biomed. Eng. 45, 525–541. 10.1007/s10439-016-1762-8 27933407 PMC6546171

[B143] Valen-Sendstad K. Mardal K.-A. Steinman D. A. (2013). High-resolution CFD detects high-frequency velocity fluctuations in bifurcation, but not sidewall, aneurysms. J. Biomech. 46, 402–407. 10.1016/j.jbiomech.2012.10.042 23174422

[B144] Valen-Sendstad K. Steinman D. A. (2014). Mind the gap: impact of computational fluid dynamics solution strategy on prediction of intracranial aneurysm hemodynamics and rupture status indicators. AJNR Am. J. Neuroradiol. 35, 536–543. 10.3174/ajnr.A3793 24231854 PMC7964722

[B145] Vignali E. Gasparotti E. Celi S. Avril S. (2021). Fully-Coupled FSI computational analyses in the ascending thoracic aorta using patient-specific conditions and anisotropic material properties. Front. Physiol. 12, 732561. 10.3389/fphys.2021.732561 34744774 PMC8564074

[B146] Wan Ab Naim W. N. Ganesan P. B. Sun Z. Chee K. H. Hashim S. A. Lim E. (2014). A perspective review on numerical simulations of hemodynamics in aortic dissection. Sci. World J. 2014, 1–12. 10.1155/2014/652520 PMC3932246 24672348

[B147] Wan Ab Naim W. N. Ganesan P. B. Sun Z. Lei J. Jansen S. Hashim S. A. (2018). Flow pattern analysis in type B aortic dissection patients after stent-grafting repair: comparison between complete and incomplete false lumen thrombosis. Int. J. Numer. Methods Biomed. Eng. 34, e2961. 10.1002/cnm.2961 29331052

[B148] Wang Q. Guo X. Brooks M. Chuen J. Poon E. K. W. Ooi A. (2022a). MRI in CFD for chronic type B aortic dissection: ready for prime time? Comput. Biol. Med. 150, 106138. 10.1016/j.compbiomed.2022.106138 36191393

[B149] Wang X. Ghayesh M. H. Kotousov A. Zander A. C. Amabili M. Dawson J. A. (2023). Biomechanics of abdominal aortic aneurysm in the framework of Windkessel effect and fully-developed inflow velocity via two-way non-linear FSI. Int. J. Non-Linear Mech. 157, 104517. 10.1016/j.ijnonlinmec.2023.104517

[B150] Wang Z. You Y. Yin Z. Bao Q. Lei S. Yu J. (2022b). Burden of aortic aneurysm and its attributable risk factors from 1990 to 2019: an analysis of the global burden of disease study 2019. Front. Cardiovasc. Med. 9, 901225. 10.3389/fcvm.2022.901225 35711350 PMC9197430

[B151] Wei W. Evin M. Rapacchi S. Kober F. Bernard M. Jacquier A. (2019). Investigating heartbeat-related in-plane motion and stress levels induced at the aortic root. Biomed. Eng. OnLine 18, 19. 10.1186/s12938-019-0632-7 30808342 PMC6391796

[B152] Wen J. Huang H. Su Z. Jiang L. Gao Q. Chen X. (2023). Predicting the risk of type B aortic dissection using hemodynamic parameters in aortic arches: a comparative study between healthy and repaired aortas. Comput. Methods Programs Biomed. 230, 107326. 10.1016/j.cmpb.2022.107326 36608431

[B153] Wen J. Yan T. Su Z. Huang H. Gao Q. Chen X. (2022). Risk evaluation of type B aortic dissection based on WSS-based indicators distribution in different types of aortic arch. Comput. Methods Programs Biomed. 221, 106872. 10.1016/j.cmpb.2022.106872 35594583

[B154] Westerhof N. Stergiopulos N. Noble M. I. M. Westerhof B. E. (2019). Snapshots of hemodynamics: an aid for clinical research and graduate education. Cham: Springer International Publishing. 10.1007/978-3-319-91932-4

[B155] Williams J. G. Marlevi D. Bruse J. L. Nezami F. R. Moradi H. Fortunato R. N. (2022). Aortic dissection is determined by specific shape and hemodynamic interactions. Ann. Biomed. Eng. 50, 1771–1786. 10.1007/s10439-022-02979-0 35943618 PMC11262626

[B156] Winter D. C. Nerem R. M. (1984). Turbulence in pulsatile flows. Ann. Biomed. Eng. 12, 357–369. 10.1007/BF02407780 6532271

[B157] Wolf I. Vetter M. Wegner I. Nolden M. Bottger T. Hastenteufel M. (2004). The medical imaging interaction toolkit (MITK): a toolkit facilitating the creation of interactive software by extending VTK and ITK. Editor GallowayJr. R. L. (San Diego, CA), 16. 10.1117/12.535112

[B158] Womersley J. R. (1957). The mathematical analysis of the arterial circulation in a state of oscillatory motion. Wright Air Dev. Cent. Tech. Rep. *WADC-TR-56-614* .

[B159] Xu H. Piccinelli M. Leshnower B. G. Lefieux A. Taylor W. R. Veneziani A. (2018). Coupled morphological-hemodynamic computational analysis of type B aortic dissection: a longitudinal study. Ann. Biomed. Eng. 46, 927–939. 10.1007/s10439-018-2012-z 29594688

[B160] Xu H. Xiong J. Han X. Mei Y. Shi Y. Wang D. (2021). Computed tomography-based hemodynamic index for aortic dissection. J. Thorac. Cardiovasc. Surg. 162, e165–e176. 10.1016/j.jtcvs.2020.02.034 32217023

[B161] Yilmaz F. (2008). “A critical review on blood flow in large arteries; relevance to blood rheology,” in Viscosity models, and physiologic conditions, 20.

[B162] Youssefi P. Gomez A. Arthurs C. Sharma R. Jahangiri M. Alberto Figueroa C. (2018). Impact of patient-specific inflow velocity profile on hemodynamics of the thoracic aorta. J. Biomech. Eng. 140. 10.1115/1.4037857 28890987

[B163] Yushkevich P. A. Gao Y. Gerig G. (2016). “ITK-SNAP: an interactive tool for semi-automatic segmentation of multi-modality biomedical images,” in 2016 38th annual international conference of the IEEE engineering in medicine and biology society (EMBC), USA, 16-20 Aug. 2016, 3342–3345. 10.1109/EMBC.2016.7591443 PMC5493443 28269019

[B164] Zambrano B. A. Gharahi H. Lim C. Jaberi F. A. Choi J. Lee W. (2016). Association of intraluminal thrombus, hemodynamic forces, and abdominal aortic aneurysm expansion using longitudinal CT images. Ann. Biomed. Eng. 44, 1502–1514. 10.1007/s10439-015-1461-x 26429788 PMC4826625

[B165] Zhang X. Mao B. Che Y. Kang J. Luo M. Qiao A. (2023). Physics-informed neural networks (PINNs) for 4D hemodynamics prediction: an investigation of optimal framework based on vascular morphology. Comput. Biol. Med. 164, 107287. 10.1016/j.compbiomed.2023.107287 37536096

[B166] Zhou S. Xu L. Hao L. Xiao H. Yao Y. Qi L. (2019). A review on low-dimensional physics-based models of systemic arteries: application to estimation of central aortic pressure. Biomed. Eng. OnLine 18, 41. 10.1186/s12938-019-0660-3 30940144 PMC6446386

[B167] Zhou Z. Teng B. Zhao Y. Wang Z. (2021). Comparison of small symptomatic and asymptomatic abdominal aortic aneurysms based on computational fluid dynamics analysis. Med. Baltim. 100, e27306. 10.1097/MD.0000000000027306 PMC8483881 34596128

[B168] Zilber Z. A. Boddu A. Malaisrie S. C. Hoel A. W. Mehta C. K. Vassallo P. (2021). Noninvasive morphologic and hemodynamic evaluation of type B aortic dissection: state of the art and future perspectives. Radiol. Cardiothorac. Imaging 3, e200456. 10.1148/ryct.2021200456 34235440 PMC8250421

