# Supplementary material for: Computational fluid dynamics modelling of hemodynamics in aortic aneurysm and dissection: a review
Source: Front Bioeng Biotechnol. 2025 Mar 21;13:1556091. doi: 10.3389/fbioe.2025.1556091 (PMC11968685; doi:10.3389/fbioe.2025.1556091)
Supplement: Supplementary file 1 [file DataSheet1.pdf]

Supplementary Material

1 Supplementary Tables

Supplementary Table 1. Proposed hemodynamic metrics to characterize hemodynamics.

Category	Metrics	Equations	Explanations	Descriptions	Ref.
Blood flow pattern	Vorticity	$\boldsymbol{\omega} = \nabla \times \mathbf{v}$	$\boldsymbol{\omega}$ is the vorticity vector, ∇ is nabla, and $\mathbf{v}$ is the velocity vector.	Flow vorticity is defined as the vector that describes flow rotation, with units of s^{-1}.	(Ramaekers et al., 2021)
	Vortex structure	$\mathbf{S} = \frac{1}{2}\left(\nabla \mathbf{v} + \nabla \mathbf{v}^{\mathrm{T}}\right)$<br>$\boldsymbol{\Omega} = \frac{1}{2}\left(\nabla \mathbf{v} - \nabla \mathbf{v}^{\mathrm{T}}\right)$<br>The eigenvalues of $\mathbf{S}^2 + \boldsymbol{\Omega}^2$: $\lambda_1 \geq \lambda_2 \geq \lambda_3$	$\mathbf{S}$ is the symmetric strain tensor, $\boldsymbol{\Omega}$ is the antisymmetric vorticity tensor, and $\mathbf{S}^2 + \boldsymbol{\Omega}^2$ is a symmetric tensor whose eigenvalues reveal the local structure of the flow field.	The vortex structure is identified using the λ_2 criterion, with the vortex region defined as a connected area where λ_2 is less than 0.	(Wild et al., 2023)
	Volume vortex fraction, surface vortex fraction	$\mathrm{vVF} = \frac{V_{\mathrm{v}}}{V_{\mathrm{total}}}$<br>$\mathrm{sVF} = \frac{S_{\mathrm{v}}}{S_{\mathrm{total}}}$<br>$Q = \frac{1}{2}\left(\lVert\boldsymbol{\Omega}\rVert^2 - \lVert\mathbf{S}\rVert^2\right)$	vVF is volume vortex fraction, V_{v} is the aneurysm volume identified by a positive Q, V_{total} is the total aneurysm volume, sVF is surface vortex fraction, and S_{v} is the surface layer area identified by a positive Q.	The vortex structure is identified using the Q criterion, with the vortex region defined as the area where Q is greater than 0. When calculating sVF, the cell-centroid variable values in the layer of elements adjacent to the wall were used.	(Varble et al., 2017)
	Vortex core line length	$\mathrm{Corelen} = \frac{1}{N}\sum_{i=1}^{N} L_i$<br>$\boldsymbol{\omega} \times \mathbf{v} = 0$	Corelen represents the average vortex core line length across N snapshots, where L_i is the vortex core line length at the *i-th* snapshot.	The vortex core lines are identified based on a collinearity condition between the instantaneous vorticity and velocity vectors.	(Byrne et al., 2014)

	Flow coherence	$\mathrm{AWCD} = \frac{1}{l}\frac{1}{N}\sum_{i=1}^{N} \mathrm{R}_i^Q \bullet s_{\mathrm{STJ}-i}$	AWCD is the average weighted curvilinear distance, where l is the length of the thoracic aorta centerline, N is the number of nodes in the computational domain, R_i^Q is the Pearson correlation coefficient calculated between flow waveform at the sinotubular junction and velocity waveform at the *i-th* node, and $s_{\mathrm{STJ}-i}$ is the curvilinear distance between the reference node at the sinotubular junction (STJ) and the *i-th* node.	AWCD can be utilized to assess the transport of flow coherence in the distal aorta. Although the definition of flow coherence is still elusive, it is strengthened here by (1) considering the flow rate waveform at the STJ as a key factor influencing hemodynamics in healthy individuals, and (2) computing the persistence length of correlation using the AWCD metric, which strategically emphasizes correlations at voxels located farther from the STJ.	(Calò et al., 2023b)
	Local normalized helicity	$\mathrm{LNH} = \frac{\mathbf{v} \cdot \boldsymbol{\omega}}{\lvert\mathbf{v}\rvert\lvert\boldsymbol{\omega}\rvert}$	LNH is the local normalized helicity. Based on this metric, several variants have been proposed, such as helical flow index (HFI), time-averaged helicity (h_1), average helicity intensity (h_2), singed helical rotation balance (h_3), unsigned helical rotation balance (h_4), dominant rotation volumetric ratio (h_5), dominant helicity ratio (h_6).	LNH is defined as the cosine of the angle between velocity and vorticity vectors, with values ranging from -1 to 1. It is used to describe the flow rotation along the centerline. A positive LNH indicates a right-handed helical pattern, while a negative LNH indicates a left-handed pattern.	(Morbiducci et al., 2011; Gallo et al., 2012)
	Inflow concentration index	$\mathrm{ICI} = \frac{Q_{\mathrm{in}}/Q_{\mathrm{v}}}{A_{\mathrm{in}}/A_{\mathrm{o}}}$	ICI is the inflow concentration index, where Q_{in} is the inflow rate into the aneurysm, Q_{v} is the flow rate in the parent artery, A_{in} is the area of the inflow region, and A_{o} is the area of the entire aneurysm orifice.	ICI is used to measure the concentration of blood flow into the aneurysm. It was first proposed for intracranial saccular aneurysms and defined as the percentage of the parent artery's flow rate into the aneurysm divided by the	(Cebral et al., 2011)

				percentage of the aneurysm ostium area corresponding to positive inflow velocity.	
Blood flow stability	Oscillatory velocity index	$\mathrm{OVI} = \frac{1}{2}\left(1 - \frac{\left\lvert\int_0^T \mathbf{v}_\mathrm{i}\,\mathrm{d}t\right\rvert}{\int_0^T \lvert\mathbf{v}_\mathrm{i}\rvert\,\mathrm{d}t}\right)$	OVI is the oscillatory velocity index, where $\mathbf{v}_\mathrm{i}$ is the instantaneous velocity vector, and T is the cardiac cycle.	OVI is used to quantify blood flow stability in the computational domain, with values ranging from 0 to 0.5. A higher OVI indicates a stronger change in the direction of the velocity vector, reflecting greater flow instability.	(Sano et al., 2017)
	Fluctuating kinetic energy	$\mathrm{FKE} = \frac{1}{2}\left(u_{rms}^2 + v_{rms}^2 + w_{rms}^2\right)$	FKE is the fluctuating kinetic energy, where u_{rms}, v_{rms} and w_{rms} are the root mean square values of the velocity fluctuations in the three directions.	FKE is mathematically equivalent to turbulence kinetic energy but considers both turbulence activity and inter-cycle variations in transitional blood flow.	(Chnafa et al., 2014; Varble et al., 2016)
	PODent, PODenum	$\mathrm{PODent} = -\sum_{i=1}^{N} P_i \ln\left(P_i\right)$<br>$P_i = \frac{\lambda_i}{\sum_{j=1}^{N}\lambda_j}$	PODent is the entropy of the energy eigenvalues of the proper orthogonal decomposition (POD) modes, where P_i is the relative energy of *i-th* mode, N is the number of modes, and λ_i is the energy eigenvalue of *i-th* mode.<br>PODenum refers to the number of POD modes account for a specific percentage of total energy, typically 95%.	POD is a dimensionality reduction technique that breaks down complex spatiotemporal flow fields into several orthogonal spatial modes and time coefficients. These spatial modes are ranked by their corresponding energy levels, with the first few models typically capturing most of the flow characteristics, while the latter mode represent small-scale details or noise.	(Byrne et al., 2014; Detmer et al., 2018)
Energy-based	Energy loss	$\mathrm{EL} = \sum_{\mathrm{inlet}}\left(p_\mathrm{i} + \frac{1}{2}\rho v_\mathrm{i}^2\right)q_\mathrm{i} - \sum_{\mathrm{outlet}}\left(p_\mathrm{o} + \frac{1}{2}\rho v_\mathrm{o}^2\right)q_\mathrm{o}$			(Qian et al., 2010, 2011)
			EL is the energy loss, where p, v, q, ρ are static pressure, velocity, flow rate and blood density, respectively. The subscripts i and o indicate the inlet and outlet, respectively.	Energy loss occurs due to flow separation, turbulence, surface friction, and flow attachment of blood in vessels, measured in watts (W). Changes in the morphology or structure of blood	

				vessels associated with cardiovascular diseases often result in abnormal energy loss.	
	Pressure loss coefficient	$\mathrm{PLc}=\dfrac{\left(p_{\mathrm{i}}+\frac{1}{2}\rho v_{\mathrm{i}}^{2}\right)-\left(p_{\mathrm{o}}+\frac{1}{2}\rho v_{\mathrm{o}}^{2}\right)}{\frac{1}{2}\rho v_{\mathrm{i}}^{2}}$			(Takao et al., 2012)
			PLc is the pressure loss coefficient.	PLc is a dimensionless quantity calculated by normalizing the energy loss with respect to the inlet kinetic energy.	
	Kinetic energy ratio	$\mathrm{KER_C}=\dfrac{\int_{V_{\mathrm{a}}}\frac{1}{2}\rho v^{2}\mathrm{d}V\Big/V_{\mathrm{a}}}{\int_{V_{\mathrm{n}}}\frac{1}{2}\rho v^{2}\mathrm{d}V\Big/V_{\mathrm{n}}}$ or $\mathrm{KER_L}=\dfrac{\int_{S_{\mathrm{l}}}\int_{T}\frac{1}{2}v^{2}\ \mathrm{dt\ ds}}{\int_{S_{\mathrm{h}}}\int_{T}\frac{1}{2}v^{2}\ \mathrm{dt\ ds}}$	$\mathrm{KER_C}$ is the kinetic energy ratio proposed by Cebral et al. (Cebral et al., 2011), where V_{a} is the volume of the aneurysm and V_{n} is the volume of the near-parent artery. $\mathrm{KER_L}$ is the kinetic energy ratio proposed by Lodi Rizzini et al. (Lodi Rizzini et al., 2024), where S_{l} and S_{h} are the surface areas of the lesional and healthy vessels, respectively.	$\mathrm{KER_c}$ was first proposed to quantify the kinetic energy content in intracranial saccular aneurysms relative to that in the near-parent artery. Obviously, its value is influenced by the choice of the near-parent artery. $\mathrm{KER_l}$ was first proposed to quantify the variation of kinetic energy in the lesion segment of the coronary artery relative to that in healthy artery.	(Cebral et al., 2011; Lodi Rizzini et al., 2024)
	Viscous dissipation ratio	$\mathrm{VDR}=\dfrac{\int_{V_{\mathrm{a}}}\mu\gamma^{2}\mathrm{d}V\Big/V_{\mathrm{a}}}{\int_{V_{\mathrm{n}}}\mu\gamma^{2}\mathrm{d}V\Big/V_{\mathrm{n}}}$ $\gamma=\sqrt{2e_{ij}e_{ij}}$ $e_{ij}=\frac{1}{2}\left(\frac{\partial u_i}{\partial x_j}+\frac{\partial u_j}{\partial x_i}\right)$	VDR is the viscous dissipation ratio, where μ is blood viscosity, γ is the strain rate, and e_{ij} is the strain rate tensor.	VDR was first proposed to quantify the amount of dissipation of mechanical energy due to viscous effects in intracranial saccular aneurysms relative to that in the near-parent artery. Obviously, its value is influenced by the choice of the near-parent artery.	(Cebral et al., 2011)
	Rotational energy ratio	$\mathrm{RER}=\dfrac{\int_{S_{\mathrm{l}}}\int_{T}\varepsilon\ \mathrm{dt\ ds}}{\int_{S_{\mathrm{h}}}\int_{T}\varepsilon\ \mathrm{dt\ ds}}$	RER is the rotational energy ratio, where ε is the scalar quantity enstrophy, and S_{l} and S_{h} are the	RER was first proposed to quantify the variation of specific rotational energy in the lesion segment of the coronary artery relative to that in healthy artery.	(Lodi Rizzini et

		$\varepsilon = \frac{1}{2}\lvert\boldsymbol{\omega}\rvert^2$	surface areas of the lesional and healthy vessels, respectively.		al., 2024)
WSS-based wall parameters	Wall shear stress	$\mathrm{WSS}=\boldsymbol{\tau}_\mathrm{w} = \mu\frac{\partial \mathbf{v}_{\parallel}}{\partial d_{\perp}}$	WSS is the wall shear stress vector, where μ is the dynamic viscosity, and $\frac{\partial \mathbf{v}_{\parallel}}{\partial d_{\perp}}$ is the gradient of the velocity parallel to the wall along the normal direction.	WSS is used to characterize the friction caused by blood flow. Its magnitude is determined by the velocity gradient and fluid viscosity, while its direction is tangential to both the direction of blood flow and the vessel surface.	(Saqr, 2019; Roux et al., 2020)
	Time-averaged wall shear stress	$\mathrm{TAWSS} = \frac{1}{T}\int_0^T \lvert\boldsymbol{\tau}_\mathrm{w}\rvert \mathrm{d}t$	TAWSS is the time-averaged WSS, where T is the cardiac cycle.	TAWSS typically refers to the time-averaged WSS magnitude over a cardiac cycle, while the time-averaged WSS vector is also used in some studies (Shimogonya et al., 2009; Arzani et al., 2014). It is important to distinguish between the two concepts.	(Hyun et al., 2000)
	Spatial wall shear stress gradient	$\mathrm{WSSGs}_{mn} = \sqrt{\left(\frac{\partial \boldsymbol{\tau}_{\mathrm{w},m}}{\partial m}\right)^2 + \left(\frac{\partial \boldsymbol{\tau}_{\mathrm{w},n}}{\partial n}\right)^2}$ $\mathrm{WSSGs}_{xyz} = \sqrt{\left(\frac{\partial \boldsymbol{\tau}_\mathrm{w}}{\partial x}\right)^2 + \left(\frac{\partial \boldsymbol{\tau}_\mathrm{w}}{\partial y}\right)^2 + \left(\frac{\partial \boldsymbol{\tau}_\mathrm{w}}{\partial z}\right)^2}$			(Tanaka et al., 2018; Sheikh et al., 2020; Lei, n.d.)
			WSSGs_{mn} is the spatial wall shear stress gradient calculated along the m and n directions, where m represents the direction of the time-averaged WSS, and n is the tangential direction to the surface, normal to m. WSSGs_{xyz} is the spatial wall shear stress gradient calculated along the Cartesian coordinates.	Both WSSGs_{mn} and WSSGs_{xyz} capture the spatial gradient of WSS, though they rely on different reference coordinates. The gradient values calculated by these methods exhibit similar distributions. Research has demonstrated that WSSGs_{mn} can impact the orientation of endothelial cells (Dolan et al., 2013; Lei, n.d.).	
	Temporal wall shear stress gradient	$\mathrm{WSSGt} = \frac{\partial \lvert\boldsymbol{\tau}_\mathrm{w}\rvert}{\partial t}$	WSSGt is the temporal wall shear stress gradient where t is the time.	WSSGt is used to describe the variation of WSS during the cardiac cycle.	(Ojha, 1994;

					Glor et al., 2005)
	Oscillatory shear index	$\mathrm{OSI}=\frac{1}{2}\left(1-\frac{\left\lvert\int_0^T \boldsymbol{\tau}_\mathrm{w}\mathrm{d}t\right\rvert}{\int_0^T\left\lvert\boldsymbol{\tau}_\mathrm{w}\right\rvert\mathrm{d}t}\right)$	OSI is the oscillatory shear index, where T is the cardiac cycle.	OSI is used to quantify the directional variation of WSS vector, with values ranging from 0 to 0.5. Regions with high OSI typically align with areas of recirculating blood flow.	(He and Ku, 1996)
	Wall shear stress vector cycle variation	$\mathrm{WSSVV}=\int_0^T \Delta\theta_t \mathrm{d}t$	WSSVV is the wall shear stress vector cycle variation, where T is the cardiac cycle, and $\Delta\theta_t$ is the directional change in WSS vector with respect to time t.	WSSVV was first proposed to identify thin-walled regions in unruptured IAs. It effectively reflects the directional changes of the WSS vector during the cardiac cycle.	(Kimura et al., 2019)
	Aneurysm formation indicator	$\mathrm{AFI}=\frac{\boldsymbol{\tau}_\mathrm{w}\cdot\int_0^T \boldsymbol{\tau}_\mathrm{w}\mathrm{d}t}{\left\lvert\boldsymbol{\tau}_\mathrm{w}\right\rvert * \left\lvert\int_0^T \boldsymbol{\tau}_\mathrm{w}\mathrm{d}t\right\rvert}$	AFI is the aneurysm formation indicator and is typically calculated at the period of mid-systolic deceleration.	AFI was first proposed to predict the locations of aneurysm formation in intracranial arteries. It essentially represents the cosine of the angle between the instantaneous WSS vector and the cycle-averaged WSS vector.	(Mantha et al., 2006)
	Cross-flow index	$\mathrm{CFI}=\frac{\boldsymbol{\tau}_\mathrm{w}}{\left\lvert\boldsymbol{\tau}_\mathrm{w}\right\rvert}\cdot\left(\mathbf{n}\times\frac{\int_0^T \boldsymbol{\tau}_\mathrm{w}\mathrm{d}t}{\left\lvert\int_0^T \boldsymbol{\tau}_\mathrm{w}\mathrm{d}t\right\rvert}\right)$	CFI is the cross-flow index, where **n** represents the unit normal vector of the vessel surface.	CFI is calculated similarly to AFI, with the key difference being that CFI represents the sine of the angle between the instantaneous WSS vector and the cycle-averaged WSS vector.	(Mohamied et al., 2017)
	Wall shear stress angle deviation	$\mathrm{WSSAD}=C\cos^{-1}\left(\frac{\int_0^T \boldsymbol{\tau}_{\mathrm{w},i}\mathrm{d}t\cdot\int_0^T \boldsymbol{\tau}_{\mathrm{w},j}\mathrm{d}t}{\left\lvert\int_0^T \boldsymbol{\tau}_{\mathrm{w},i}\mathrm{d}t\right\rvert\cdot\left\lvert\int_0^T \boldsymbol{\tau}_{\mathrm{w},j}\mathrm{d}t\right\rvert}\right)$ $C=\begin{cases}1.0; & \left(\frac{1}{T}\int_0^T \mathbf{v}_\mathrm{n}\mathrm{d}t\right)\cdot\mathbf{n}_i \geq 0 \\ 0.0; & \left(\frac{1}{T}\int_0^T \mathbf{v}_\mathrm{n}\mathrm{d}t\right)\cdot\mathbf{n}_i < 0\end{cases}$			(Hyun et al., 2000)

			WSSAD is the wall shear stress angle deviation, where $\boldsymbol{\tau}_{\mathrm{w},i}$ is the WSS vector at surface point i, $\boldsymbol{\tau}_{\mathrm{w},j}$ is the WSS vector at neighboring cell j, $\mathbf{v}_{\mathrm{n}}$ is the near-wall velocity component normal to the surface i, and $\mathbf{n}_i$ is the normal vector of the surface i.	WSSAD was proposed to quantify the directional differences of WSS vectors on the vessel wall at a regional scale. Elevated WSSAD was expected to signify regions prone to thrombotic particle aggregation and wall deposition. It should be noted that the value of WSSAD is related to the computational mesh.	
	Wall shear stress angle gradient	$\mathrm{WSSAG} = \frac{1}{T}\int_0^T \left\lvert \frac{1}{A_i}\int_S \nabla\varphi \mathrm{d}A_i \right\rvert \mathrm{d}t$ $\varphi = \cos^{-1}\left(\frac{\boldsymbol{\tau}_{\mathrm{w},i}\cdot\boldsymbol{\tau}_{\mathrm{w},j}}{\lvert\boldsymbol{\tau}_{\mathrm{w},i}\rvert\cdot\lvert\boldsymbol{\tau}_{\mathrm{w},j}\rvert}\right)$			(Longest and Kleinstreuer, 2000; Goubergrits et al., 2008)
			WSSAG is the wall shear stress angle gradient, where A_i is the surface area of control volume.	WSSAG is the time-averaged magnitude of the angle gradient for the control volume surface area A_i. Large WSSAG values appeared to co-localize with regions of dysfunctional cells and intimal thickening. Differentiation and integration operations amplify the impact of uncertainties on the noise in results.	
	Endothelial cell activation potential	$\mathrm{ECAP} = \frac{\mathrm{OSI}}{\mathrm{TAWSS}}$	ECAP is the endothelial cell activation potential, where OSI is the oscillatory shear index, and TAWSS is the time-averaged wall shear stress.	ECAP was proposed to characterize the degree of 'thrombogenic susceptibility'. A high ECAP means the region is exposed to a low/oscillatory WSS. Some researchers considered 1.4 Pa^{-1} as the critical threshold of ECAP for intraluminal thrombus formation (Kelsey et al., 2017; Deyranlou et al., 2020).	(Di Achille et al., 2014)

	Transverse wall shear stress	$\mathrm{transWSS}=\frac{1}{T}\int_0^T\left\|\boldsymbol{\tau}_{\mathrm{w}}\cdot\left(\mathbf{n}\times\frac{\int_0^T\boldsymbol{\tau}_{\mathrm{w}}\mathrm{d}t}{\left\|\int_0^T\boldsymbol{\tau}_{\mathrm{w}}\mathrm{d}t\right\|}\right)\right\|\mathrm{d}t$			(Peiffer et al., 2013)
			TransWSS is the transverse wall shear stress, where **n** represents the unit normal vector of the vessel surface.	TransWSS was proposed to evaluate the multidirectional characteristic of the WSS vector throughout the cardiac cycle. However, transWSS cannot differentiate between purely forward and reversing unidirectional flows, as both scenarios yield a transWSS value of zero. The value of transWSS ranges from 0 to TAWSS.	
	Local WSS vector projections along the axial and secondary directions	$\mathrm{WSS}_{\mathrm{ax}}=\frac{\boldsymbol{\tau}_{\mathrm{w}}\cdot\mathbf{C}}{\|\mathbf{C}\|}\frac{\mathbf{C}}{\|\mathbf{C}\|}$ $\mathrm{WSS}_{\mathrm{sc}}=\frac{\boldsymbol{\tau}_{\mathrm{w}}\cdot\mathbf{S}}{\|\mathbf{S}\|}\frac{\mathbf{S}}{\|\mathbf{S}\|}$ $\mathbf{S}=\frac{\mathbf{C}\times\mathbf{R}}{\|\mathbf{C}\|\|\mathbf{R}\|}$			(Morbiducci et al., 2015)
			$\mathrm{WSS}_{\mathrm{ax}}$ and $\mathrm{WSS}_{\mathrm{sc}}$ are the projections of WSS vector along the axial and secondary directions, respectively. $\mathbf{C}$ is the unit centerline vector, $\mathbf{R}$ is the unit vector directed from the point of application on the centerline to the point of interest, and $\mathbf{S}$ is the external product of $\mathbf{C}$ and $\mathbf{R}$.	This projection approach, applied along the axial and secondary directions, was proposed to characterize the multidirectional nature of the WSS vector. In this method, the preferential direction is the tangential direction of the vascular centerline, rather than the direction perpendicular to the cycle-averaged WSS vector as defined in transWSS.	

	Gradient oscillatory number	$\mathrm{GON} = 1 - \dfrac{\left\lvert \int_0^T \mathbf{G} \mathrm{d}t \right\rvert}{\int_0^T \lvert \mathbf{G} \rvert \mathrm{d}t}$ $\mathbf{G} = \left(\dfrac{\partial \boldsymbol{\tau}_{\mathrm{w},m}}{\partial m}, \dfrac{\partial \boldsymbol{\tau}_{\mathrm{w},n}}{\partial n} \right)$			(Shimogonya et al., 2009)
			GON is the gradient oscillatory number, where T is the cardiac cycle, $\mathbf{G}$ is the WSSGs vector, and $\boldsymbol{\tau}_{\mathrm{w},m}$ and $\boldsymbol{\tau}_{\mathrm{w},n}$ are WSS components along m and n directions, respectively.	GON was proposed to quantify the degree of oscillating tension and compression forces to evaluate IA formation, with values ranging from 0 to 1. Lower GON values indicate a less transition between tension and compression, while higher values suggest greater occurrences of such transitions.	
	Wall shear stress divergence	$\mathrm{WSSD} = \dfrac{\partial \boldsymbol{\tau}_{\mathrm{w},x}}{\partial x} + \dfrac{\partial \boldsymbol{\tau}_{\mathrm{w},y}}{\partial y} + \dfrac{\partial \boldsymbol{\tau}_{\mathrm{w},z}}{\partial z}$			(Zhang et al., 2013)
			WSSD is the wall shear stress divergence, where $\boldsymbol{\tau}_{\mathrm{w},x}$, $\boldsymbol{\tau}_{\mathrm{w},y}$, and $\boldsymbol{\tau}_{\mathrm{w},y}$ are WSS components along the x, y, and z directions, respectively.	WSSD was proposed to characterize the stretching or compressing on the vessel surface for the evaluation of IA rupture. Positive values of WSSD represent blood stretching the aneurysm surface, whereas negative values indicate a compression effect.	
	Topological shear variation index	$\mathrm{TSVI} = \left\{ \dfrac{1}{T} \int_0^T \left[\mathrm{DIV}_{\mathrm{WSS}} - \overline{\mathrm{DIV}_{\mathrm{WSS}}} \right]^2 \mathrm{d}t \right\}^{1/2}$ $\mathrm{DIV}_{\mathrm{WSS}} = \nabla \cdot \left(\dfrac{\boldsymbol{\tau}_{\mathrm{w}}}{\lvert \boldsymbol{\tau}_{\mathrm{w}} \rvert} \right)$			(De Nisco et al., 2020; Morbiducci et al., 2020)
			TSVI is the topological shear variation index, where T is the cardiac cycle, and $\mathrm{DIV}_{\mathrm{WSS}}$ is the divergence of the unit WSS vector.	TSVI was proposed to measure the variability in WSS contraction/expansion exerted at the vessel wall. High TSVI values indicate	

				greater variability in WSS contraction and expansion, which can lead to fluctuations in intra- and intercellular tension, potentially influencing disease progression.	
	Relative residence time	$\mathrm{RRT}=\frac{1}{(1-2\times \mathrm{OSI})\times \mathrm{TAWSS}}=\frac{1}{\frac{1}{T}\left\lvert\int_0^T \boldsymbol{\tau}_{\mathrm{w}}\mathrm{d}t\right\rvert}$			(Himburg et al., 2004)
			RRT is the relative residence time, where OSI is the oscillatory shear index, and TAWSS is the time-averaged wall shear stress.	RRT was proposed to qualitatively assess the residence time of the solutes and formed elements in the blood. It is independent of physical time and is expressed in units of Pa^{-1}. Areas with higher RRT correspond to circulation and stagnation zones with lower TAWSS and higher OSI.	
	WSS exposure time and WSS residence time	$\mathrm{WSS}_{\mathrm{ET}}(e)=\sqrt{\frac{A_m}{A_e}}\sum_{p=1}^{N_t}\int_0^T H_e(p,t)\mathrm{d}t$ $H_e=\begin{cases}1; & \text{if } x_p(t)\in e\\ 0; & \text{if } x_p(t)\notin e\end{cases}$ $\mathrm{WSS}_{\mathrm{RT}}(\mathbf{x}_0,t_0;\mathit{\Gamma})=\min(t)\in(0,T)\ \text{ s.t. } \mathrm{x}(\mathbf{x}_0,t_0+t)\notin \mathit{\Gamma}$			(Arzani et al., 2016)
			$\mathrm{WSS}_{\mathrm{ET}}$ and $\mathrm{WSS}_{\mathrm{RT}}$ are the wall shear stress exposure and residence time, respectively. A_e and A_m are the area of the element and the average area of all the elements, respectively. x_p is the position of the near wall trajectory, H_e is the indicator function for element *e*, N_t is the total number of trajectories, T	$\mathrm{WSS}_{\mathrm{ET}}$ and $\mathrm{WSS}_{\mathrm{RT}}$ were proposed based on Lagrangian wall shear stress structures. $\mathrm{WSS}_{\mathrm{ET}}$ characterizes the intensity of species accumulation on the vessel wall, whereas $\mathrm{WSS}_{\mathrm{RT}}$ measures the time required for species to escape the near-wall region. The threshold of the near wall domain was chosen within the concentration boundary layer	

			is the integration time, and Γ is the near wall flow domain.	thickness. Compared to RRT, $\mathrm{WSS_{ET}}$ can provide a better estimation of the near-wall stagnation and accumulation of the chemicals in complex flows, as evidenced by the stronger correlations between the $\mathrm{WSS_{ET}}$ and surface concentrations.	
	Activation potential	$\mathrm{AP}\left(\mathbf{x}_0, t_0; t\right)=\int_{t_0}^{t}\left\lVert\mathbf{e}\left(\mathbf{x}, t\right)\right\rVert_F \,\mathrm{d}t$ $\left\lVert\mathbf{e}\right\rVert_F=\sqrt{\sum_{i=1}^{3}\sum_{j=1}^{3} e_{ij}^2}$ $e_{ij}=\frac{1}{2}\left(\frac{\partial u_i}{\partial x_j}+\frac{\partial u_j}{\partial x_i}\right)$	AP is the activation potential for a platelet at position $\mathbf{x}_0=\mathbf{x}\left(t_0\right)$ at time t_0, where e_{ij} is the strain rate tensor, $\left\lVert\mathbf{e}\right\rVert_F$ is the Frobenius norm of $\mathbf{e}$, and t and t_0 are the final and initial moments of the particle tracking, respectively.	AP is a dimensionless scalar metric that represents the magnitude of shear stress accumulated by a particle during the tracked period. While the mechanism of platelet activation is not fully understood, platelets with higher AP values are generally considered more likely to be activated than those with lower values.	(Shadden and Hendabadi, 2013)
	Thrombus formation potential	$\mathrm{TFP}=\mathrm{ECAP}\cdot\mathrm{AP}=\frac{\mathrm{OSI}\cdot\mathrm{AP}}{\mathrm{TAWSS}}$	TFP is the thrombus formation potential, where ECAP is the endothelial cell activation potential, AP is the activation potential for platelets, OSI is the oscillatory shear index, and TAWSS is the time-averaged wall shear stress.	TFP was proposed to identify potential thrombus formation sites by combining information on the flow-induced shear history experienced by blood-borne particles near the endothelium with data on both the time-averaged wall shear stress and the oscillatory shear index, which locally influence endothelial mechanobiology.	(Di Achille et al., 2014)
	Dominant harmonic of WSS	$\mathrm{DH}=\max\left(F_{\lvert\tau_{\mathrm{w}}\rvert}\left(n\frac{2\pi}{T}\right)\right),\quad n\in\mathbf{N}^{+}$			(Himburg and Friedman, 2006)
			DH is the dominant harmonic, which is the harmonic with the highest amplitude in the Fourier decomposition of the time-varying WSS magnitude. $F_{\lvert\tau_{\mathrm{w}}\rvert}$ is the Fourier-transform of the time-	DH was proposed by converting the WSS history experienced by the blood vessel wall from the time domain to the frequency domain. This approach establishes a connection between blood flow and the frequency-based response	

			varying of the WSS magnitude and *n* is a positive integer.	of endothelial cells. However, its applicability may be limited in complex flow patterns with significant nonaxial blood flow (Lee et al., 2009).	
	Harmonic index of WSS	$\mathrm{HI}=\frac{\sum_{n=1}^{\infty}F_{\lvert\boldsymbol{\tau}_{\mathrm{w}}\rvert}\left(n\frac{2\pi}{T}\right)}{\sum_{n=0}^{\infty}F_{\lvert\boldsymbol{\tau}_{\mathrm{w}}\rvert}\left(n\frac{2\pi}{T}\right)},\quad n\in\mathbf{N}^{+}$			(Gelf and et al., 2006)
			HI is the harmonic index, where $F_{\lvert\boldsymbol{\tau}_{\mathrm{w}}\rvert}$ is the Fourier-transform of the time-varying of the WSS magnitude and *n* is a positive integer.	HI was proposed to measure the relative contributions of the dynamic and static components of the WSS signal. Its values range from 0 to 1, where 0 indicates a steady, non-zero WSS signal, and 1 represents a purely oscillatory signal with a time-averaged value of zero.	
	Spectral power index of WSS	$\mathrm{SPI}=\frac{\sum_{n=n_{\mathrm{c}}}^{\infty}\left\lvert F_{\lvert\boldsymbol{\tau}_{\mathrm{w}}\rvert}\left(n\frac{2\pi}{T}\right)\right\rvert^{2}}{\sum_{n=1}^{\infty}\left\lvert F_{\lvert\boldsymbol{\tau}_{w}\rvert}\left(n\frac{2\pi}{T}\right)\right\rvert^{2}},\quad n\in\mathbf{N}^{+}$			(Khan et al., 2017)
			SPI is the spectral power index, where $F_{\lvert\boldsymbol{\tau}_{\mathrm{w}}\rvert}$ is the Fourier-transform of the time-varying of the WSS magnitude, *n* is a positive integer, and n_c is the harmonic corresponding to the cut-off frequency.	SPI was proposed to quantify the high-frequency instabilities of WSS. It is a normalized quantity ranging from 0 to 1, where 0 indicates no flow instability and 1 indicates complete flow instability.	
Metrics in the FSI simulation	Wall displacement	$\mathbf{D}=\left(\Delta\mathbf{x},\Delta\mathbf{y},\Delta\mathbf{z}\right)$	**D** is the wall displacement, with the components $\Delta\mathbf{x}$, $\Delta\mathbf{y}$, and $\Delta\mathbf{z}$ corresponding to displacements along the *x*, *y*, and *z* coordinates, respectively. In FSI modeling, vessel walls are	The heterogeneity of vascular wall materials makes it challenging to accurately predict deformation in FSI simulations. An alternative approach, the moving-boundary method, uses wall	(Calò et al., 2023a)

			considered compliant rather than rigid, as assumed in traditional CFD simulations. This allows for the analysis of wall displacement during the cardiac cycle.	displacements from CTA images to directly specify vascular geometry changes and predict hemodynamics.	
	Von mises stress	$\mathrm{VMS}=\sqrt{\frac{1}{2}\left[\left(\sigma_{xx}-\sigma_{yy}\right)^{2}+\left(\sigma_{yy}-\sigma_{zz}\right)^{2}+\left(\sigma_{zz}-\sigma_{xx}\right)^{2}\right]+3\left(\tau_{xy}^{2}+\tau_{yz}^{2}+\tau_{zx}^{2}\right)}$			(Schoenborn et al., 2024)
			VMS is the von mises stress, where σ is the principal stresses in different directions and τ is the shear stresses in different directions.	VMS simplifies the three-dimensional stress into an equivalent stress, facilitating comparison with the yield stress of the vessel wall to assess the risk of rupture.	
	Rupture potential index	$\mathrm{RPI}=\frac{\sigma_{\mathrm{VMS}}}{\sigma_{\mathrm{ultimate}}}$<br>$\sigma_{\mathrm{ultimate}}=719-379\times\left(\sqrt{\mathrm{ILT}}-0.81\right)-156\times\left(\mathrm{NORD}-2.46\right)-213\times\mathrm{HIST}+193\times\mathrm{SEX}\left[\mathrm{kPa}\right]$			(Vande Geest et al., 2006; Maier et al., 2010)
			RPI is the rupture potential index, where σ_{VMS} is the von mises stress, $\sigma_{\mathrm{ultimate}}$ is the ultimate stress, also known as wall strength, ILT is the local intraluminal thrombus thickness, ranging from 0 to 3.6 cm, NORD is a normalized diameter, ranging from 1.06 to 3.9, HIST equals 0.5 if a first-degree relative has had an abdominal aortic aneurysm and -0.5 otherwise, and SEX equals 0.5 if male and -0.5 if female.	RPI, defined as the ratio of wall stress to wall strength, was proposed to quantify the risk of aneurysm rupture. When the stress on the vessel wall exceeds its strength, the risk of rupture increases. The calculation of wall strength for an aneurysm is typically based on empirical models and may be influenced by factors such as local thrombus thickness, local aneurysm diameter, family history of aneurysms, and gender. Besides von Mises stress, the calculation can also employ the first principal Cauchy stress as the denominator.	

Supplementary Table 2. Summary of hemodynamic studies on aortic aneurysms.

**Ref.**	**Pathology**	***n***	**CFD modeling**	**Objective**	**Main finding about hemodynamics**
Les et al. (2010)	AAA	8	Method: parallel; Imaging method: MRA; Wall: rigid and no-slip; Inlet: patient-specific flow rate with Womersley profile; Outlet: three-element Windkessel model; Viscosity model: Newtonian model; Turbulence model: DNS; Time dependance: transient; Convergence criteria: NA; Statistical convergence: five cycles after the first three cardiac cycles; Hemodynamic metrics: velocity, normal stresses, WSS, time-averaged WSS vector, OSI, turbulent kinetic energy; Medical imaging software: Geodesic; CFD software: NA.	To examine how exercise impacts AAA hemodynamics and its potential role in slowing AAA growth.	Exercise led to increased WSS and turbulence and decreased OSI in AAA, which may attenuate AAA growth.
Suh et al. (2011)	AAA	10	Method: parallel; Imaging method: MRA; Wall: rigid and no-slip; Inlet: patient-specific flow rate with Womersley profile; Outlet: three-element Windkessel model; Viscosity model: Newtonian model; Turbulence model: NA; Time dependance: transient; Convergence criteria: NA; Statistical convergence: the fifth cardiac cycle; Hemodynamic metrics: time-averaged WSS vector, OSI, particle residence time, particle residence index, half-life time; Medical imaging software: Geodesic; CFD software: NA.	To quantify the impact of lower-limb exercise intensity on hemodynamics in patients with AAA.	Increased activity levels resulted in higher WSS and lower OSI and particle residence time.
Arzani et al. (2014)	AAA	10	Method: longitudinal; Imaging method: MRA; Wall: rigid and no-slip; Inlet: patient-specific flow rate with Womersley profile; Outlet: three-element Windkessel model; Viscosity model: Newtonian model; Turbulence model: DNS; Time dependance: transient; Convergence criteria: NA; Statistical convergence: four cycles after the first two cardiac cycles; Hemodynamic metrics: TAWSS, OSI; Medical imaging software: SimVascular; CFD software: NA.	To explore the relationship between hemodynamic metrics and progression of thrombus inside AAA.	Low OSI regions correlated strongly with ILT growth, while high OSI (>0.4) and low TAWSS (<1 dyn/cm²) areas did not match thrombus locations.
Boyd et al. (2016)	AAA	8	Method: parallel; Imaging method: CTA; Wall: rigid and no-slip; Inlet: 5 L/min with Poiseuille profile; Outlet:	To explore the relationship between	AAA rupture tends to occur in areas of

			NA; Viscosity model: Newtonian model; Turbulence model: laminar; Time dependance: steady; Convergence criteria: 10^{-6}; Statistical convergence: NA; Hemodynamic metrics: velocity, pressure, WSS; Medical imaging software: Mimics; CFD software: OpenFOAM.	hemodynamic characteristics and AAA rupture.	blood recirculation, characterized by low WSS and thrombus deposition.
Zambrano et al. (2016)	AAA	14	Method: longitudinal; Imaging method: CTA; Wall: rigid and no-slip; Inlet: empirical flow rate; Outlet: empirical pressure waveform; Viscosity model: Carreau-Yasuda non-Newtonian model; Turbulence model: laminar; Time dependance: transient; Convergence criteria: NA; Statistical convergence: the third cardiac cycle; Hemodynamic metrics: TAWSS; Medical imaging software: Mimics; CFD software: Ansys Fluent.	To better understand the mechanisms by which hemodynamic forces contribute to ILT accumulation and AAA expansion.	Low WSS may promote ILT accumulation.
Chisci et al. (2018)	AAA	143	Method: parallel; Imaging method: CTA; Wall: rigid and no-slip; Inlet: NA; Outlet: NA; Viscosity model: Newtonian model; Turbulence model: NA; Time dependance: transient; Convergence criteria: NA; Statistical convergence: NA; Hemodynamic metrics: OSI, TAWSS, RRT; Medical imaging software: NA; CFD software: ElmerSolver.	To develop a scoring system for grading AAA rupture risk.	OSI from 2D CFD simulations and TAWSS from 3D CFD simulations can predict AAA rupture risk.
Qiu et al. (2019)	AAA	13	Method: parallel; Imaging method: CTA; Wall: rigid and no-slip; Inlet: empirical flow rate; Outlet: three-element Windkessel model; Viscosity model: Newtonian model; Turbulence model: laminar; Time dependance: transient; Convergence criteria: 10^{-5} for momentum, 10^{-3} for continuity; Statistical convergence: the fifth cardiac cycle; Hemodynamic metrics: flow pattern, TAWSS, $WSSGs_{xyz}$, OSI, ECAP; Medical imaging software: Mimics; CFD software: Ansys Fluent.	To explore the impact of ILT on hemodynamics in ruptured AAA.	Recirculation flow and low WSS may negatively impact local rupture or provide protection by promoting the formation of thin-layered ILT.
Joly et al. (2020)	AAA	41	Method: longitudinal, parallel; Imaging method: CTA; Wall: rigid and no-slip; Inlet: empirical flow rate with Womersley profile; Outlet: three-element Windkessel model; Viscosity model: Quemada non-Newtonian model; Turbulence model: laminar; Time dependance:	To explore the relationship between morphology, hemodynamic	$TAWSS_{min}$, RRT_{mean}, $ECAP_{max,mean,stdev}$ could distinguish between healthy groups, low risk

			transient; Convergence criteria: 10^{-6} for pressure and 10^{-8} for velocity; Statistical convergence: after 5-7 cardiac cycles; Hemodynamic metrics: TAWSS, OSI, RRT, ECAP; Medical imaging software: ITK-SNAP; CFD software: OpenFOAM.	metrics, and AAA growth.	groups, and high risk groups.
Meyrignac et al. (2020)	AAA	81	Method: longitudinal, parallel; Imaging method: CECT; Wall: NA; Inlet: empirical flow rate with parabolic profile; Outlet: percentage flow rate and zero pressure; Viscosity model: Newtonian model; Turbulence model: NA; Time dependance: transient; Convergence criteria: NA; Statistical convergence: NA; Hemodynamic metrics: wall pressure, TAWSS; Medical imaging software: OsiriX or TeraRecon; CFD software: Yales2Bio.	To explore the relationship between volumetric, hemodynamic metrics, and AAA growth.	High WSS within an AAA appeared to act as a protective factor for growth rate.
Bappoo et al. (2021)	AAA	295	Method: longitudinal, parallel; Imaging method: CTA; Wall: rigid and no-slip; Inlet: empirical flow rate; Outlet: three-element Windkessel model; Viscosity model: Carreau-Yasuda non-Newtonian model; Turbulence model: NA; Time dependance: transient; Convergence criteria: NA; Statistical convergence: five cardiac cycles; Hemodynamic metrics: TAWSS; Medical imaging software: 3D Slicer; CFD software: STAR-CCM+.	To examine the impact of baseline low shear stress at on AAA expansion rate and future aneurysm-related events.	Baseline low shear stress (<0.4 Pa) was linked to AAA expansion and future aneurysm-related events.
Zhou et al. (2021)	AAA	86	Method: parallel; Imaging method: CTA; Wall: rigid and no-slip; Inlet: 0.18 m/s, 140 mmHg; Outlet: 0 mmHg; Viscosity model: Newtonian model; Turbulence model: NA; Time dependance: steady; Convergence criteria: NA; Statistical convergence: NA; Hemodynamic metrics: WSS; Medical imaging software: Mimics; CFD software: Ansys Fluent.	To compare hemodynamic parameters of symptomatic and asymptomatic AAAs to identify risk factors for rupture.	WSS in the symptomatic group was lower than in the asymptomatic group.
McClarty et al. (2022)	ATAA	5	Method: NA; Imaging method: MRA; Wall: rigid and no-slip; Inlet: patient-specific velocity profile; Outlet: three-element Windkessel model; Viscosity model: Newtonian model; Turbulence model: laminar; Time	To explore the effect of aortic hemodynamics on	High WSS at the wall of ATAA was linked to local degradation of arterial wall

			dependance: transient; Convergence criteria: NA; Statistical convergence: the sixth cardiac cycle; Hemodynamic metrics: WSS, TAWSS, OSI, RRT; Medical imaging software: ITK-SNAP; CFD software: SimVascular.	arterial wall properties in ATAA.	viscoelastic hysteresis and delamination strength, serving as a surrogate for aortic dissection.
Qiu et al. (2022)	AAA	106	Method: parallel; Imaging method: CTA; Wall: rigid and no-slip; Inlet: 0.1 m/s; Outlet: zero pressure; Viscosity model: Newtonian model; Turbulence model: laminar; Time dependance: steady; Convergence criteria: NA; Statistical convergence: NA; Hemodynamic metrics: WSS, area of low WSS, pressure drop, impingement pressure increase, flow patterns (flow impingement, vortex structure, helical flow); Medical imaging software: Mimics; CFD software: Ansys Fluent.	To assess the predictive value of flow patterns derived from CFD simulations in AAA rupture.	A helical main flow channel with helical vortices was linked to increased AAA rupture risk. Incorporating flow patterns may improve the detection of high-risk aneurysms.
Teng et al. (2022)	AAA	35	Method: parallel; Imaging method: CTA; Wall: nonlinear, isotropic, hyperelastic material; Inlet: 0.8 m/s; Outlet: 140 mmHg; Viscosity model: Newtonian model; Turbulence model: NA; Time dependance: transient; Convergence criteria: NA; Statistical convergence: NA; Hemodynamic metrics: WSS; Medical imaging software: Mimics; CFD software: Ansys Fluent.	To explore the effects of geometric and hemodynamic parameters on AAA rupture.	High diameter and curvature values, along with low WSS, distinguished patients at high risk for AAA rupture.
Salmasi et al. (2023)	ATAA	33	Method: NA; Imaging method: 4D-flow MRI; Wall: rigid and no-slip; Inlet: patient-specific velocity profile; Outlet: three-element Windkessel model; Viscosity model: Carreau-Yasuda non-Newtonian model; Turbulence model: SST turbulence model; Time dependance: transient; Convergence criteria: NA; Statistical convergence: NA; Hemodynamic metrics: WSS, TAWSS; Medical imaging software: Mimics; CFD software: Ansys CFX.	To explore the relationship between geometry and flow in a cohort of patients with ATAA.	Accelerated velocity and higher WSS may predict ATAA prognosis.
Ramaekers et al. (2024)	TAA	8	Method: parallel; Imaging method: 4D-flow MRI; Wall: rigid and no-slip; Inlet: patient-specific flow rate with plug profile; Outlet: three-element Windkessel model;	To explore the hemodynamic characteristics in	Helicity and vorticity were lower in the TAA patient, while

			Viscosity model: Carreau-Yasuda non-Newtonian model; Turbulence model: laminar; Time dependance: transient; Convergence criteria: 10^{-3} for flow and pressure; Statistical convergence: the fifth cardiac cycle; Hemodynamic metrics: velocity, WSS, TAWSS, OSI, ECAP, vorticity, helicity, vortex structure by the *Q* criterion; Medical imaging software: CAAS MR Solutions and VMTK; CFD software: Ansys Fluent.	TAA and the impact of hypertension on hemodynamics in TAA.	ECAP was higher. The effect of hypertension on hemodynamic parameters was not significant.
Rezaeitaleshmahalleh et al. (2024)	AAA	70	Method: parallel; Imaging method: CTA; Wall: rigid and no-slip; Inlet: empirical flow rate; Outlet: zero pressure; Viscosity model: Newtonian model; Turbulence model: laminar, LES; Time dependance: transient; Convergence criteria: 10^{-3} for velocity and continuity; Statistical convergence: the fourth cardiac cycle; Hemodynamic metrics: WSS, OSI, vortex structure, turbulent kinetic energy; Medical imaging software: Mimics; CFD software: Ansys Fluent..	To compare the performance of the laminar flow model and LES in predicting hemodynamics.	The hemodynamic metrics calculated from the laminar and LES simulations demonstrated similar effectiveness in distinguishing the growth status of AAAs.

AAA: abdominal aortic aneurysm, MRA: magnetic resonance angiography, DNS: direct numerical simulation, NA: not available, WSS: wall shear stress, OSI: oscillatory shear index, TAWSS: time-averaged wall shear stress, ILT: intraluminal thrombus, CTA: computed tomography angiography, CECT: contrast-enhanced computed tomography, RRT: relative residence time, CFD: computational fluid dynamics, $WSSGs_{xyz}$: spatial wall shear stress gradient calculated along the Cartesian coordinates, ECAP: endothelial cell activation potential, ATAA: ascending thoracic aortic aneurysm, MRI: magnetic resonance imaging, TAA: thoracic aortic aneurysm, VMTK: vascular modeling toolkit, LES: large eddy simulation.

Supplementary Table 3. Summary of hemodynamic studies on aortic dissections.

**Ref.**	**Pathology**	***n***	**CFD modeling**	**Objective**	**Main finding about hemodynamics**
Cheng et al. (2013)	TBAD	5	Method: parallel, longitudinal; Imaging method: CTA; Wall: rigid and no-slip; Inlet: empirical flow rate; Outlet: 0 Pa; Viscosity model: Newtonian model; Turbulence model: SST turbulence model; Time dependance: transient; Convergence criteria: 10^{-6}; Statistical convergence: the third cardiac cycle; Hemodynamic metrics: TAWSS, RRT; Medical imaging software: Mimics; CFD software: Ansys CFX.	To explore the impact of aortic and primary tear morphology on flow characteristics and clinical outcomes in patients with acute TBAD.	Aortic morphology, as well as the size and position of the primary entry tear, significantly influence flow and other hemodynamic parameters in TBAD.
Shang et al. (2015)	TBAD	14	Method: parallel, longitudinal; Imaging method: CTA; Wall: rigid and no-slip; Inlet: empirical flow rate; Outlet: zero pressure; Viscosity model: Newtonian model; Turbulence model: NA; Time dependance: transient; Convergence criteria: 10^{-6}; Statistical convergence: the third cardiac cycle; Hemodynamic metrics: velocity, TAWSS; Medical imaging software: Amira; CFD software: Abaqus/CFD.	To identify the risk of subacute or chronic aneurysmal dilation of TBAD patients.	In patients with rapidly expanding aneurysms, the total flow rate through the false lumen and TAWSS were significantly higher.
Chi et al. (2017)	TAAD	7	Method: parallel, virtual repair; Imaging method: CECT; Wall: rigid and no-slip; Inlet: 0.2 m/s, empirical flow rate; Outlet: steady pressure, pulsatile pressure; Viscosity model: NA; Turbulence model: SST turbulence model; Time dependance: steady, transient; Convergence criteria: NA; Statistical convergence: NA; Hemodynamic metrics: velocity, pressure, WSS; Medical imaging software: Simpleware ScanIP; CFD software: Ansys Fluent.	To explore the relationship between WSS and frequent tearing locations in TAAD.	TAAD exhibits higher WSS and stronger helical flow in the distal aortic arch, which may be linked to tears in this region.
Hohri et al. (2021)	TAAD	6	Method: parallel; Imaging method: CECT; Wall: rigid and no-slip; Inlet: empirical flow rate; Outlet: pulsative pressure; Viscosity model: Newtonian model; Turbulence model: RNG k-epsilon turbulence model; Time dependance: transient; Convergence criteria: 10^{-5}; Statistical convergence: NA; Hemodynamic metrics: velocity, WSS, OSI; Medical imaging software: OsiriX and 3D-Coat; CFD software: Ansys Fluent.	To clarify the hemodynamic mechanism of TAAD using CFD simulations.	High OSI areas with vortex flow are closely associated with the future primary entry site in TAAD.

Marrocco-Trischitta and Sturla (2022)	TBAD	15	Method: parallel; Imaging method: CTA; Wall: rigid and no-slip; Inlet: empirical flow rate; Outlet: three-element Windkessel model; Viscosity model: Newtonian model; Turbulence model: NA; Time dependance: transient; Convergence criteria: NA; Statistical convergence: the sixth cardiac cycle; Hemodynamic metrics: velocity, vorticity, LNH; Medical imaging software: Mimics; CFD software: LifeV.	To investigate whether type III arches, known a high incidence of TBAD, exhibit a consistent secondary helical flow pattern.	The type III arch configuration was linked to a specific, consistent abnormal secondary helical flow pattern, potentially explaining its high prevalence in TBAD patients.
Wen et al. (2022)	TBAD	30	Method: parallel; Imaging method: CTA; Wall: rigid and no-slip; Inlet: empirical flow rate with flat profile; Outlet: three-element Windkessel model; Viscosity model: Carreau-Yasuda non-Newtonian model; Turbulence model: NA; Time dependance: transient; Convergence criteria: 10^{-7}; Statistical convergence: the fifth cardiac cycle; Hemodynamic metrics: TAWSS, OSI, RRT, transWSS, CFI; Medical imaging software: Mimics; CFD software: Ansys Fluent.	To explore hemodynamic differences across aortic arch types and their impact on TBAD occurrence.	Moste indicators, like TAWSS, OSI, and RRT, were similar across aortic arch types, but maximum CFI correlated positively with type III aortic arch in proximal descending aorta.
Williams et al. (2022)	TAAD, ATAA	31	Method: longitudinal, parallel; Imaging method: CTA; Wall: rigid and no-slip; Inlet: empirical flow rate; Outlet: zero pressure; Viscosity model: Newtonian model; Turbulence model: laminar; Time dependance: transient; Convergence criteria: NA; Statistical convergence: the third cardiac cycle; Hemodynamic metrics: WSS; Medical imaging software: CAAS MR Solutions and VMTK; CFD software: Ansys CFX.	To explore geometric and hemodynamic contributors to TAAD among patients with ATTA.	Higher maximum WSS in the ascending aorta was linked to an increased risk of dissection.
Li et al. (2023)	TBAD	27	Method: parallel, virtual repair; Imaging method: CTA; Wall: rigid and no-slip; Inlet: empirical flow rate; Outlet: three-element Windkessel model; Viscosity model: Newtonian model; Turbulence model: laminar; Time dependance: transient; Convergence criteria: NA; Statistical convergence: NA; Hemodynamic metrics: TAWSS, OSI, transWSS normalized by TAWSS, helicity;	To explore the role of flow features and hemodynamic parameters in indicating the risk of TBAD occurrence.	Contralateral helical flow was lost in the aortic arch and descending aorta in TBAD patients. TBAD patients exhibited higher normalized transWSS, with PET

			Medical imaging software: Mimics; CFD software: Ansys Fluent.		locations overlapping high transWSS areas.
Wen et al. (2023)	TBAD	16	Method: parallel, virtual repair; Imaging method: CTA; Wall: rigid and no-slip; Inlet: empirical flow rate with flat profile; Outlet: three-element Windkessel model; Viscosity model: Carreau-Yasuda non-Newtonian model; Turbulence model: NA; Time dependance: transient; Convergence criteria: 10^{-7}; Statistical convergence: the fifth cardiac cycle; Hemodynamic metrics: TAWSS, OSI, CFI, TSVI, LNH, h_1, h_2; Medical imaging software: Mimics; CFD software: Ansys Fluent.	To investigate hemodynamic parameter variations in aortas prior to TBAD onset.	Hemodynamic variations, like elevated OSI and CFI, may better predict TBAD risk and tear locations than anatomical features.

TBAD: type-B aortic dissection, CTA: computed tomography angiography, CECT: contrast-enhanced computed tomography, TAWSS: time-averaged wall shear stress, RRT: relative residence time, NA: not available, TAAD: type-A aortic dissection, SST: shear stress transport, WSS: wall shear stress, RNG: re-normalization group, OSI: oscillatory shear index, CFD: computational fluid dynamics, LNH: local normalized helicity, transWSS: transverse wall shear stress, CFI: cross-flow index, ATAA: ascending thoracic aortic aneurysm, VMTK: vascular modeling toolkit, TSVI: topological shear variation index, h_1: time-averaged helicity, h_2: average helicity intensity.

2 Supplementary Material — Systematic search strategy

We conducted a systematic search to investigate the application of CFD-based hemodynamic modeling in aortic aneurysm and aortic dissection. To ensure the significance of certain hemodynamic features, we focused on studies with a study population of five or more. The systematic search was performed on the PubMed database for articles published online before 23 October 2024, with titles or abstracts containing the following keywords:

- aortic aneurysm* OR aortic dissection
- hemodynamic* OR haemodynamic* OR shear
- computational fluid dynamics OR CFD

We screened the 298 identified articles based on the following inclusion and exclusion criteria.

Inclusion criteria:

- The article was original.
- The study was based on patient-specific modeling, meaning the artery geometry was anatomically realistic.
- The article was in English.

Exclusion criteria:

- The subject was animal or cadaveric.
- The study was in vitro.
- The article was incomplete or withdrawn, or there was no access to the full text.
- The article or data was duplicated.
- The study focused on the therapeutic effects of medical devices, such as stents and coils.
- The number of cases included in the study was fewer than five.

Two independent reviewers evaluated these articles for inclusion. Following the screening process, as shown in **Supplementary Figure 1**, a total of 26 articles were included for analysis.

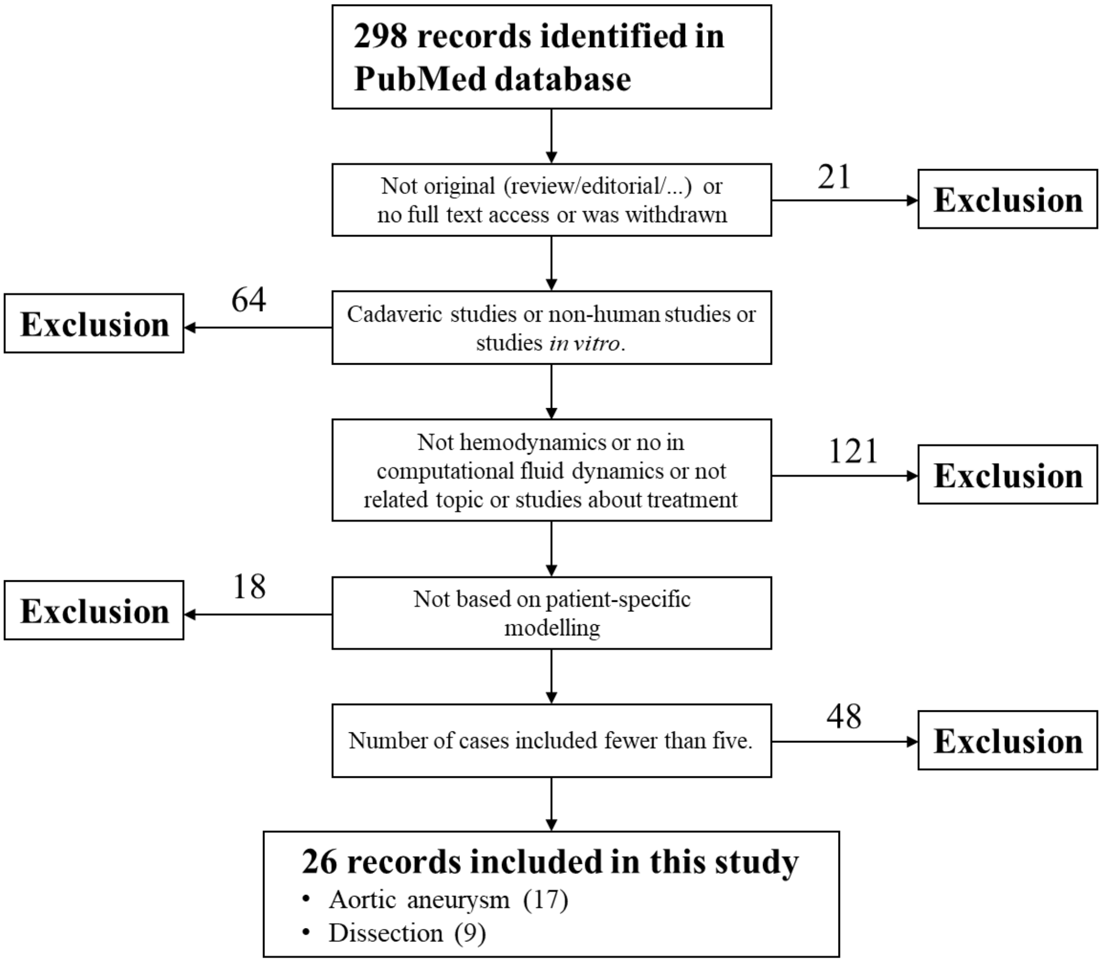

Supplementary Figure 1. Literature selection process based on specific inclusion and exclusion criteria.

References

Arzani, A., Gambaruto, A. M., Chen, G., and Shadden, S. C. (2016). Lagrangian wall shear stress structures and near-wall transport in high-Schmidt-number aneurysmal flows. *J. Fluid Mech.* 790, 158–172. doi: 10.1017/jfm.2016.6

Arzani, A., Suh, G.-Y., Dalman, R. L., and Shadden, S. C. (2014). A longitudinal comparison of hemodynamics and intraluminal thrombus deposition in abdominal aortic aneurysms. *Am. J. Physiol.-Heart Circ. Physiol.* 307, H1786–H1795. doi: 10.1152/ajpheart.00461.2014

Bappoo, N., Syed, M. B. J., Khinsoe, G., Kelsey, L. J., Forsythe, R. O., Powell, J. T., et al. (2021). Low Shear Stress at Baseline Predicts Expansion and Aneurysm-Related Events in Patients With Abdominal Aortic Aneurysm. *Circ. Cardiovasc. Imaging* 14, 1112–1121. doi: 10.1161/CIRCIMAGING.121.013160

Boyd, A. J., Kuhn, D. C. S., Lozowy, R. J., and Kulbisky, G. P. (2016). Low wall shear stress predominates at sites of abdominal aortic aneurysm rupture. *J. Vasc. Surg.* 63, 1613–1619. doi: 10.1016/j.jvs.2015.01.040

Byrne, G., Mut, F., and Cebral, J. (2014). Quantifying the Large-Scale Hemodynamics of Intracranial Aneurysms. *AJNR Am. J. Neuroradiol.* 35, 333–338. doi: 10.3174/ajnr.A3678

Calò, K., Capellini, K., De Nisco, G., Mazzi, V., Gasparotti, E., Gallo, D., et al. (2023a). Impact of wall displacements on the large-scale flow coherence in ascending aorta. *J. Biomech.* 154, 111620. doi: 10.1016/j.jbiomech.2023.111620

Calò, K., Gallo, D., Guala, A., Rizzini, M. L., Dux-Santoy, L., Rodriguez-Palomares, J., et al. (2023b). Network-Based Characterization of Blood Large-Scale Coherent Motion in the Healthy Human Aorta With 4D Flow MRI. *IEEE Trans. Biomed. Eng.* 70, 1095–1104. doi: 10.1109/TBME.2022.3209736

Cebral, J. R., Mut, F., Weir, J., and Putman, C. (2011). Quantitative characterization of the hemodynamic environment in ruptured and unruptured brain aneurysms. *AJNR Am. J. Neuroradiol.* 32, 145–151. doi: 10.3174/ajnr.A2419

Cheng, Z., Riga, C., Chan, J., Hamady, M., Wood, N. B., Cheshire, N. J. W., et al. (2013). Initial findings and potential applicability of computational simulation of the aorta in acute type B dissection. *J. Vasc. Surg.* 57, 35S-43S. doi: 10.1016/j.jvs.2012.07.061

Chi, Q., He, Y., Luan, Y., Qin, K., and Mu, L. (2017). Numerical analysis of wall shear stress in ascending aorta before tearing in type A aortic dissection. *Comput. Biol. Med.* 89, 236–247. doi: 10.1016/j.compbiomed.2017.07.029

Chisci, E., Alamanni, N., Iacoponi, F., Michelagnoli, S., Procacci, T., Colombo, G., et al. (2018). Grading abdominal aortic aneurysm rupture risk. *J. Cardiovasc. Surg. (Torino)* 59, 87–94. doi: 10.23736/S0021-9509.16.08848-0

Chnafa, C., Mendez, S., and Nicoud, F. (2014). Image-based large-eddy simulation in a realistic left heart. *Comput. Fluids* 94, 173–187. doi: 10.1016/j.compfluid.2014.01.030

De Nisco, G., Tasso, P., Calò, K., Mazzi, V., Gallo, D., Condemi, F., et al. (2020). Deciphering ascending thoracic aortic aneurysm hemodynamics in relation to biomechanical properties. *Med. Eng. Phys.* 82, 119–129. doi: 10.1016/j.medengphy.2020.07.003

Detmer, F. J., Chung, B. J., Mut, F., Slawski, M., Hamzei-Sichani, F., Putman, C., et al. (2018). Development and internal validation of an aneurysm rupture probability model based on patient characteristics and aneurysm location, morphology, and hemodynamics. *Int. J. Comput. Assist. Radiol. Surg.* 13, 1767–1779. doi: 10.1007/s11548-018-1837-0

Deyranlou, A., Naish, J. H., Miller, C. A., Revell, A., and Keshmiri, A. (2020). Numerical Study of Atrial Fibrillation Effects on Flow Distribution in Aortic Circulation. *Ann. Biomed. Eng.* 48, 1291–1308. doi: 10.1007/s10439-020-02448-6

Di Achille, P., Tellides, G., Figueroa, C. A., and Humphrey, J. D. (2014). A haemodynamic predictor of intraluminal thrombus formation in abdominal aortic aneurysms. *Proc. R. Soc. Math. Phys. Eng. Sci.* 470, 20140163. doi: 10.1098/rspa.2014.0163

Dolan, J. M., Kolega, J., and Meng, H. (2013). High wall shear stress and spatial gradients in vascular

pathology: a review. *Ann. Biomed. Eng.* 41, 1411–1427. doi: 10.1007/s10439-012-0695-0

Gallo, D., Steinman, D. A., Bijari, P. B., and Morbiducci, U. (2012). Helical flow in carotid bifurcation as surrogate marker of exposure to disturbed shear. *J. Biomech.* 45, 2398–2404. doi: 10.1016/j.jbiomech.2012.07.007

Gelfand, B. D., Epstein, F. H., and Blackman, B. R. (2006). Spatial and spectral heterogeneity of time-varying shear stress profiles in the carotid bifurcation by phase-contrast MRI. *J. Magn. Reson. Imaging* 24, 1386–1392. doi: 10.1002/jmri.20765

Glor, F. P., Ariff, B., Hughes, A. D., Verdonck, P. R., Thom, S. A. M., Barratt, D. C., et al. (2005). Operator dependence of 3-D ultrasound-based computational fluid dynamics for the carotid bifurcation. *IEEE Trans. Med. Imaging* 24, 451–456. doi: 10.1109/tmi.2005.844173

Goubergrits, L., Kertzscher, U., Schöneberg, B., Wellnhofer, E., Petz, C., and Hege, H.-C. (2008). CFD analysis in an anatomically realistic coronary artery model based on non-invasive 3D imaging: comparison of magnetic resonance imaging with computed tomography. *Int. J. Cardiovasc. Imaging* 24, 411–421. doi: 10.1007/s10554-007-9275-z

He, X., and Ku, D. N. (1996). Pulsatile Flow in the Human Left Coronary Artery Bifurcation: Average Conditions. *J. Biomech. Eng.* 118, 74–82. doi: 10.1115/1.2795948

Himburg, H. A., and Friedman, M. H. (2006). Correspondence of Low Mean Shear and High Harmonic Content in the Porcine Iliac Arteries. *J. Biomech. Eng.* 128, 852–856. doi: 10.1115/1.2354211

Himburg, H. A., Grzybowski, D. M., Hazel, A. L., LaMack, J. A., Li, X.-M., and Friedman, M. H. (2004). Spatial comparison between wall shear stress measures and porcine arterial endothelial permeability. *Am. J. Physiol.-Heart Circ. Physiol.* 286, H1916–H1922. doi: 10.1152/ajpheart.00897.2003

Hohri, Y., Numata, S., Itatani, K., Kanda, K., Yamazaki, S., Inoue, T., et al. (2021). Prediction for future occurrence of type A aortic dissection using computational fluid dynamics. *Eur. J. Cardio-Thorac. Surg. Off. J. Eur. Assoc. Cardio-Thorac. Surg.* 60, 384–391. doi: 10.1093/ejcts/ezab094

Hyun, S., Kleinstreuer, C., and Archie, J. P. (2000). Hemodynamics analyses of arterial expansions with implications to thrombosis and restenosis. *Med. Eng. Phys.* 22, 13–27. doi: 10.1016/S1350-4533(00)00006-0

Joly, F., Soulez, G., Lessard, S., Kauffmann, C., and Vignon-Clementel, I. (2020). A Cohort Longitudinal Study Identifies Morphology and Hemodynamics Predictors of Abdominal Aortic Aneurysm Growth. *Ann. Biomed. Eng.* 48, 606–623. doi: 10.1007/s10439-019-02375-1

Kelsey, L. J., Powell, J. T., Norman, P. E., Miller, K., and Doyle, B. J. (2017). A comparison of hemodynamic metrics and intraluminal thrombus burden in a common iliac artery aneurysm. *Int. J. Numer. Methods Biomed. Eng.* 33. doi: 10.1002/cnm.2821

Khan, M. O., Chnafa, C., Gallo, D., Molinari, F., Morbiducci, U., Steinman, D. A., et al. (2017). On the quantification and visualization of transient periodic instabilities in pulsatile flows. *J. Biomech.* 52, 179–182. doi: 10.1016/j.jbiomech.2016.12.037

Kimura, H., Taniguchi, M., Hayashi, K., Fujimoto, Y., Fujita, Y., Sasayama, T., et al. (2019). Clear Detection of Thin-Walled Regions in Unruptured Cerebral Aneurysms by Using Computational Fluid Dynamics. *World Neurosurg.* 121, e287–e295. doi: 10.1016/j.wneu.2018.09.098

Lee, S.-W., Antiga, L., and Steinman, D. A. (2009). Correlations Among Indicators of Disturbed Flow at the Normal Carotid Bifurcation. *J. Biomech. Eng.* 131. doi: 10.1115/1.3127252

Lei, M. (n.d.). Computational fluid dynamics analyses and optimal design of bifurcating blood vessels. Available at: https://www.proquest.com/docview/304267995/abstract/7F9C7CE644BC4A50PQ/1 (Accessed October 20, 2023).

Les, A. S., Shadden, S. C., Figueroa, C. A., Park, J. M., Tedesco, M. M., Herfkens, R. J., et al. (2010). Quantification of hemodynamics in abdominal aortic aneurysms during rest and exercise using magnetic resonance imaging and computational fluid dynamics. *Ann. Biomed. Eng.* 38, 1288–1313. doi: 10.1007/s10439-010-9949-x

Li, D., Wang, J., Zeng, W., Zeng, X., Liu, Z., Cao, H., et al. (2023). The loss of helical flow in the thoracic aorta might be an identifying marker for the risk of acute type B aortic dissection. *Comput. Methods Programs Biomed.* 230, 107331. doi: 10.1016/j.cmpb.2022.107331

Lodi Rizzini, M., Candreva, A., Mazzi, V., Pagnoni, M., Chiastra, C., Aben, J.-P., et al. (2024). Blood Flow Energy Identifies Coronary Lesions Culprit of Future Myocardial Infarction. *Ann. Biomed. Eng.* 52, 226–238. doi: 10.1007/s10439-023-03362-3

Longest, P. W., and Kleinstreuer, C. (2000). Computational haemodynamics analysis and comparison study of arterio-venous grafts. *J. Med. Eng. Technol.* 24, 102–110. doi: 10.1080/03091900050135013

Maier, A., Gee, M. W., Reeps, C., Pongratz, J., Eckstein, H.-H., and Wall, W. A. (2010). A Comparison of Diameter, Wall Stress, and Rupture Potential Index for Abdominal Aortic Aneurysm Rupture Risk Prediction. *Ann. Biomed. Eng.* 38, 3124–3134. doi: 10.1007/s10439-010-0067-6

Mantha, A., Karmonik, C., Benndorf, G., Strother, C., and Metcalfe, R. (2006). Hemodynamics in a Cerebral Artery before and after the Formation of an Aneurysm. *AJNR Am. J. Neuroradiol.* 27, 1113–1118.

Marrocco-Trischitta, M. M., and Sturla, F. (2022). Blood flow helical pattern in type III arch configuration as a potential risk factor for type B aortic dissection. *Eur. J. Cardiothorac. Surg.* 61, 132–139. doi: 10.1093/ejcts/ezab307

McClarty, D., Ouzounian, M., Tang, M., Eliathamby, D., Romero, D., Nguyen, E., et al. (2022). Ascending aortic aneurysm haemodynamics are associated with aortic wall biomechanical properties. *Eur. J. Cardio-Thorac. Surg. Off. J. Eur. Assoc. Cardio-Thorac. Surg.* 61, 367–375. doi: 10.1093/ejcts/ezab471

Meyrignac, O., Bal, L., Zadro, C., Vavasseur, A., Sewonu, A., Gaudry, M., et al. (2020). Combining Volumetric and Wall Shear Stress Analysis from CT to Assess Risk of Abdominal Aortic Aneurysm Progression. *Radiology* 295, 722–729. doi: 10.1148/radiol.2020192112

Mohamied, Y., Sherwin, S. J., and Weinberg, P. D. (2017). Understanding the fluid mechanics behind transverse wall shear stress. *J. Biomech.* 50, 102–109. doi: 10.1016/j.jbiomech.2016.11.035

Morbiducci, U., Gallo, D., Cristofanelli, S., Ponzini, R., Deriu, M. A., Rizzo, G., et al. (2015). A rational approach to defining principal axes of multidirectional wall shear stress in realistic vascular geometries, with application to the study of the influence of helical flow on wall shear stress directionality in aorta. *J. Biomech.* 48, 899–906. doi: 10.1016/j.jbiomech.2015.02.027

Morbiducci, U., Mazzi, V., Domanin, M., De Nisco, G., Vergara, C., Steinman, D. A., et al. (2020). Wall Shear Stress Topological Skeleton Independently Predicts Long-Term Restenosis After Carotid Bifurcation Endarterectomy. *Ann. Biomed. Eng.* 48, 2936–2949. doi: 10.1007/s10439-020-02607-9

Morbiducci, U., Ponzini, R., Rizzo, G., Cadioli, M., Esposito, A., Montevecchi, F. M., et al. (2011). Mechanistic insight into the physiological relevance of helical blood flow in the human aorta: an in vivo study. *Biomech. Model. Mechanobiol.* 10, 339–355. doi: 10.1007/s10237-010-0238-2

Ojha, M. (1994). Wall shear stress temporal gradient and anastomotic intimal hyperplasia. *Circ. Res.* 74, 1227–1231. doi: 10.1161/01.RES.74.6.1227

Peiffer, V., Sherwin, S. J., and Weinberg, P. D. (2013). Computation in the rabbit aorta of a new metric – the transverse wall shear stress – to quantify the multidirectional character of disturbed blood flow. *J. Biomech.* 46, 2651–2658. doi: 10.1016/j.jbiomech.2013.08.003

Qian, Y., Liu, J. L., Itatani, K., Miyaji, K., and Umezu, M. (2010). Computational Hemodynamic Analysis in Congenital Heart Disease: Simulation of the Norwood Procedure. *Ann. Biomed. Eng.* 38, 2302–2313. doi: 10.1007/s10439-010-9978-5

Qian, Y., Takao, H., Umezu, M., and Murayama, Y. (2011). Risk Analysis of Unruptured Aneurysms Using Computational Fluid Dynamics Technology: Preliminary Results. *Am. J. Neuroradiol.* 32, 1948–1955. doi: 10.3174/ajnr.A2655

Qiu, Y., Wang, J., Zhao, J., Wang, T., Zheng, T., and Yuan, D. (2022). Association Between Blood Flow Pattern and Rupture Risk of Abdominal Aortic Aneurysm Based on Computational Fluid Dynamics. *Eur. J. Vasc. Endovasc. Surg. Off. J. Eur. Soc. Vasc. Surg.* 64, 155–164. doi: 10.1016/j.ejvs.2022.05.027

Qiu, Y., Wang, Y., Fan, Y., Peng, L., Liu, R., Zhao, J., et al. (2019). Role of intraluminal thrombus in abdominal aortic aneurysm ruptures: A hemodynamic point of view. *Med. Phys.* 46, 4263–4275. doi: 10.1002/mp.13658

Ramaekers, M. J. F. G., Adriaans, B. P., Juffermans, J. F., van Assen, H. C., Bekkers, S. C. A. M., Scholte, A. J. H. A., et al. (2021). Characterization of Ascending Aortic Flow in Patients With Degenerative Aneurysms: A 4D Flow Magnetic Resonance Study. *Invest. Radiol.* 56, 494. doi: 10.1097/RLI.0000000000000768

Ramaekers, M. J. F. G., van der Vlugt, I. B., Westenberg, J. J. M., Perinajová, R., Lamb, H. J., Wildberger, J. E., et al. (2024). Flow patterns in ascending aortic aneurysms: Determining the

role of hypertension using phase contrast magnetic resonance and computational fluid dynamics. *Comput. Biol. Med.* 172, 108310. doi: 10.1016/j.compbiomed.2024.108310

Rezaeitaleshmahalleh, M., Lyu, Z., Mu, N., Wang, M., Zhang, X., Rasmussen, T. E., et al. (2024). Computational Hemodynamics-Based Growth Prediction for Small Abdominal Aortic Aneurysms: Laminar Simulations Versus Large Eddy Simulations. *Ann. Biomed. Eng.* doi: 10.1007/s10439-024-03572-3

Roux, E., Bougaran, P., Dufourcq, P., and Couffinhal, T. (2020). Fluid Shear Stress Sensing by the Endothelial Layer. *Front. Physiol.* 11, 861. doi: 10.3389/fphys.2020.00861

Salmasi, M. Y., Pirola, S., Mahuttanatan, S., Fisichella, S. M., Sengupta, S., Jarral, O. A., et al. (2023). Geometry and flow in ascending aortic aneurysms are influenced by left ventricular outflow tract orientation: Detecting increased wall shear stress on the outer curve of proximal aortic aneurysms. *J. Thorac. Cardiovasc. Surg.* 166, 11-21.e1. doi: 10.1016/j.jtcvs.2021.06.014

Sano, T., Ishida, F., Tsuji, M., Furukawa, K., Shimosaka, S., and Suzuki, H. (2017). Hemodynamic Differences Between Ruptured and Unruptured Cerebral Aneurysms Simultaneously Existing in the Same Location: 2 Case Reports and Proposal of a Novel Parameter Oscillatory Velocity Index. *World Neurosurg.* 98, 868.e5-868.e10. doi: 10.1016/j.wneu.2016.12.047

Saqr, K. M. (2019). Wall shear stress in the Navier-Stokes equation: A commentary. *Comput. Biol. Med.* 106, 82–83. doi: 10.1016/j.compbiomed.2019.01.012

Schoenborn, S., Pirola, S., Woodruff, M. A., and Allenby, M. C. (2024). Fluid-Structure Interaction Within Models of Patient-Specific Arteries: Computational Simulations and Experimental Validations. *IEEE Rev. Biomed. Eng.* 17, 280–296. doi: 10.1109/RBME.2022.3215678

Shadden, S. C., and Hendabadi, S. (2013). Potential fluid mechanic pathways of platelet activation. *Biomech. Model. Mechanobiol.* 12, 467–474. doi: 10.1007/s10237-012-0417-4

Shang, E. K., Nathan, D. P., Fairman, R. M., Bavaria, J. E., Gorman, R. C., Gorman, J. H., et al. (2015). Use of computational fluid dynamics studies in predicting aneurysmal degeneration of acute type B aortic dissections. *J. Vasc. Surg.* 62, 279–284. doi: 10.1016/j.jvs.2015.02.048

Sheikh, M. A. A., Shuib, A. S., and Mohyi, M. H. H. (2020). A review of hemodynamic parameters in cerebral aneurysm. *Interdiscip. Neurosurg.* 22, 100716. doi: 10.1016/j.inat.2020.100716

Shimogonya, Y., Ishikawa, T., Imai, Y., Matsuki, N., and Yamaguchi, T. (2009). Can temporal fluctuation in spatial wall shear stress gradient initiate a cerebral aneurysm? A proposed novel hemodynamic index, the gradient oscillatory number (GON). *J. Biomech.* 42, 550–554. doi: 10.1016/j.jbiomech.2008.10.006

Suh, G.-Y., Les, A. S., Tenforde, A. S., Shadden, S. C., Spilker, R. L., Yeung, J. J., et al. (2011). Hemodynamic changes quantified in abdominal aortic aneurysms with increasing exercise intensity using mr exercise imaging and image-based computational fluid dynamics. *Ann. Biomed. Eng.* 39, 2186–2202. doi: 10.1007/s10439-011-0313-6

Takao, H., Murayama, Y., Otsuka, S., Qian, Y., Mohamed, A., Masuda, S., et al. (2012). Hemodynamic

Differences Between Unruptured and Ruptured Intracranial Aneurysms During Observation. *Stroke* 43, 1436–1439. doi: 10.1161/STROKEAHA.111.640995

Tanaka, K., Takao, H., Suzuki, T., Fujimura, S., Uchiyama, Y., Otani, K., et al. (2018). Relationship between hemodynamic parameters and cerebral aneurysm initiation., in *2018 40th Annual International Conference of the IEEE Engineering in Medicine and Biology Society (EMBC)*, (Honolulu, HI: IEEE), 1347–1350. doi: 10.1109/EMBC.2018.8512466

Teng, B., Zhou, Z., Zhao, Y., and Wang, Z. (2022). Combined Curvature and Wall Shear Stress Analysis of Abdominal Aortic Aneurysm: An Analysis of Rupture Risk Factors. *Cardiovasc. Intervent. Radiol.* 45, 752–760. doi: 10.1007/s00270-022-03140-z

Vande Geest, J. P., Wang, D. H. J., Wisniewski, S. R., Makaroun, M. S., and Vorp, D. A. (2006). Towards A Noninvasive Method for Determination of Patient-Specific Wall Strength Distribution in Abdominal Aortic Aneurysms. *Ann. Biomed. Eng.* 34, 1098–1106. doi: 10.1007/s10439-006-9132-6

Varble, N., Trylesinski, G., Xiang, J., Snyder, K., and Meng, H. (2017). Identification of vortex structures in a cohort of 204 intracranial aneurysms. *J. R. Soc. Interface* 14, 20170021. doi: 10.1098/rsif.2017.0021

Varble, N., Xiang, J., Lin, N., Levy, E., and Meng, H. (2016). Flow Instability Detected by High-Resolution Computational Fluid Dynamics in Fifty-Six Middle Cerebral Artery Aneurysms. *J. Biomech. Eng.* 138, 0610091–06100911. doi: 10.1115/1.4033477

Wen, J., Huang, H., Su, Z., Jiang, L., Gao, Q., Chen, X., et al. (2023). Predicting the Risk of Type B Aortic Dissection Using Hemodynamic Parameters in Aortic Arches: A Comparative Study between Healthy and Repaired Aortas. *Comput. Methods Programs Biomed.* 230, 107326. doi: 10.1016/j.cmpb.2022.107326

Wen, J., Yan, T., Su, Z., Huang, H., Gao, Q., Chen, X., et al. (2022). Risk evaluation of type B aortic dissection based on WSS-based indicators distribution in different types of aortic arch. *Comput. Methods Programs Biomed.* 221, 106872. doi: 10.1016/j.cmpb.2022.106872

Wild, N. C., Bulusu, K. V., and Plesniak, M. W. (2023). Vortical Structures Promote Atheroprotective Wall Shear Stress Distributions in a Carotid Artery Bifurcation Model. *Bioengineering* 10, 1036. doi: 10.3390/bioengineering10091036

Williams, J. G., Marlevi, D., Bruse, J. L., Nezami, F. R., Moradi, H., Fortunato, R. N., et al. (2022). Aortic Dissection is Determined by Specific Shape and Hemodynamic Interactions. *Ann. Biomed. Eng.* 50, 1771–1786. doi: 10.1007/s10439-022-02979-0

Zambrano, B. A., Gharahi, H., Lim, C., Jaberi, F. A., Choi, J., Lee, W., et al. (2016). Association of Intraluminal Thrombus, Hemodynamic Forces, and Abdominal Aortic Aneurysm Expansion Using Longitudinal CT Images. *Ann. Biomed. Eng.* 44, 1502–1514. doi: 10.1007/s10439-015-1461-x

Zhang, Y., Takao, H., Murayama, Y., and Qian, Y. (2013). Propose a Wall Shear Stress Divergence to Estimate the Risks of Intracranial Aneurysm Rupture. *Sci. World J.* 2013, 508131. doi:

10.1155/2013/508131

Zhou, Z., Teng, B., Zhao, Y., and Wang, Z. (2021). Comparison of small symptomatic and asymptomatic abdominal aortic aneurysms based on computational fluid dynamics analysis. *Medicine (Baltimore)* 100, e27306. doi: 10.1097/MD.0000000000027306
